# Anchoring quartet-based phylogenetic distances and applications to species tree reconstruction

**DOI:** 10.1186/s12864-016-3098-z

**Published:** 2016-11-11

**Authors:** Erfan Sayyari, Siavash Mirarab

**Affiliations:** Department of Electrical and Computer Engineering, University of California, San Diego, 9500 Gilman Dr, La Jolla CA, 92093 USA

**Keywords:** Incomplete lineage sorting, Quartet methods, Multi-species coalescent, Species tree

## Abstract

**Background:**

Inferring species trees from gene trees using the coalescent-based summary methods has been the subject of much attention, yet new scalable and accurate methods are needed.

**Results:**

We introduce DISTIQUE, a new statistically consistent summary method for inferring species trees from gene trees under the coalescent model. We generalize our results to arbitrary phylogenetic inference problems; we show that two arbitrarily chosen leaves, called anchors, can be used to estimate relative distances between all other pairs of leaves by inferring relevant quartet trees. This results in a family of distance-based tree inference methods, with running times ranging between quadratic to quartic in the number of leaves.

**Conclusions:**

We show in simulated studies that DISTIQUE has comparable accuracy to leading coalescent-based summary methods and reduced running times.

**Electronic supplementary material:**

The online version of this article (doi:10.1186/s12864-016-3098-z) contains supplementary material, which is available to authorized users.

## Background

The evolutionary histories of species and genes can be discordant [1], necessitating a distinction between genes trees and species trees. Incomplete Lineage Sorting (ILS), modeled by the multi-species coalescent (MSC) model [2], is one of the main causes of discordance. A fast approach for estimating the species relationships in the face of such discordances is to first estimate a gene tree for each gene and to summarize the gene trees to build a species tree. The summary method, thus, takes as input a set of gene trees and returns a species tree. A desirable property for a summary method is statistical consistency (a theoretical guarantee that it converges in probability to the correct species tree as the number of error-free genes increases). Many statistically consistent summary methods are available (e.g., ASTRAL [3, 4], BUCKy-population [5], and MP-EST [6]), and coalescent-based species tree estimation is a vibrant field of research, with many recent examples of successful biological analyses [7–9] (see [10–14] for criticism of these methods, especially their sensitivity to gene tree error).

Inferring trees using pairwise distances is a well-studied general method of phylogenetic reconstruction [15–18], and several summary methods are distance-based. These methods first compute a pairwise distance between *species* based on input gene trees and then use a distance method (e.g., neighbor joining [17]) to build the species tree; examples of distance-based summary methods are STAR [19], GLASS [20], NJst [21], and its new implementation, ASTRID [22].

Another powerful general approach to phylogenetic reconstruction is analyzing quartets, which are subsets of four leaves in a tree. Quartet methods first infer a set of quartet trees and then combine them to build a tree on the full dataset [16, 23, 24]. Induced quartet trees have also been used [24–28] to combine a collection of input trees to build a so-called supertree [29]. Quartet-based phylogeny estimation has been revived in recent years [3, 5, 30–32] because of its connections to coalescent-based analyses [33–35]. Under the MSC model, for unrooted species trees with four leaves, the most likely unrooted gene tree is identical to the species tree [33] (but this is not true for larger trees [34, 36]). Furthermore, the length of the internal branch in a quartet species tree (in coalescent units) defines the probabilities of the three possible gene tree quartet topologies [34]. Some recent and statistically consistent quartet-based species tree estimation methods rely on these results. For example, ASTRAL seeks the species tree with the maximum number of quartet trees shared with input gene trees [3, 4].

In this paper, we introduce a new coalescent-based summary method, called DISTIQUE (Distance-based Inference of Species Trees from Induced QUartet Elements). Like ASTRAL, DISTIQUE is based on quartets, but instead of directly optimizing a quartet score, it uses quartets to compute pairwise distances, which are then used as input to a distance method. The innovative aspect of DISTIQUE is its method of calculating distances. It chooses two arbitrary “anchor” species and computes the frequency of quartet trees induced by gene trees that include the two anchors as sisters. We show that these frequencies can be transformed into an asymptotically additive distance matrix; using this matrix with a consistent distance-based method (e.g., neighbor joining) gives a statistically consistent summary method. This method would generate a species tree on all species except the two anchors in *Θ*(*n* ^2^ *k*) (for *n* species and *k* gene trees). However, using multiple anchor pairs can increase accuracy and can ensure all species are included in the final tree. Various strategies for choosing anchors and combining their results are introduced, with running times ranging between *Θ*(*n* ^2^ *k*) and *Θ*(*n* ^4^ *k*).

After describing DISTIQUE, we show that the anchoring approach can be generalized to any tree inference problem. Assume we have a way to compute the topology and the internal branch length for any quartet of leaves. We show that as long as this quartet estimator is consistent, our anchoring mechanism and a certain family of transformations can be used to compute an additive distance matrix, which in turn can be used to infer the correct tree topology but not correct branch lengths. This result is rather surprising because, for any pair of anchors and a pair of other leaves, the quartet internal branch length will often be very different from the distance between non-anchor leaves. Thus, anchoring produces incorrect pairwise distances that are nevertheless additive for the correct tree topology. DISTIQUE uses anchoring because for the MSC-based species tree inference, pairwise species distances are not straightforward to define but inferring quartet trees is easy. We evaluate the accuracy of DISTIQUE on simulated and biological data and show that its accuracy is competitive with the best alternative methods even when used with relatively small subsets of all possible anchors.

## Methods

**Notation and background:** Let $\mathcal {L}$ denote the leaf-set of size *n*. For an unrooted tree *T* on $\mathcal {L}$, the set of quartet trees induced on all possible ${n \choose 4}$ quartets of leaves is denoted by $\mathcal {Q}^{T}$. We use *a* *b*.*c* *d* to denote that *a* and *b* are sisters in the quartet tree on {*a*,*b*,*c*,*d*}. A tree *T* is equivalent to a distance matrix *D* ^*T*^, computed by summing lengths of the edges between pairs of leaves, and a distance matrix that corresponds to a tree is called additive [37]. We refer to the unique tree [37] associated with the additive distance matrix *D* as *T* ^*D*^ or *T*. Also, $T|\mathcal {L}'$ and $D|\mathcal {L}'$ denote *T* and *D* restricted to the leaf-set $\mathcal {L}'$.

To test for the additivity of a distance matrix *D*, we can use the four point condition [37]. For a quartet of leaves $Q=\{a,b,c,d\}\subset \mathcal {L}$, the median and the maximum of the following three values should be the same: {*D*[*a*,*b*]+*D*[*c*,*d*],*D*[*a*,*c*]+*D*[*b*,*d*],*D*[*a*,*d*]+*D*[*b*,*c*]}. When internal branch lengths are assumed positive, as we do throughout this paper, the minimum value is strictly smaller than the median. Assuming w.l.o.g. *D*[*a*,*b*]+*D*[*c*,*d*] is the smallest value, we can infer *a* *b*.*c* *d* is the topology induced by *T* ^*D*^. Let *τ*(*Q*)>0 denote the length of the single internal branch in this quartet tree, which we call its “quartet length”; i.e., if $ab.cd \in \mathcal {Q}^{T}$, then $\tau (Q)= \frac {1}{2} (D[a,c] + D[b,d] - D[a,b] - D[c,d])$.

### General theoretical results

#### **Definition 1**

Given two positive constants *α*,*β*and a monotonically increasing function *f*(*x*) bounded above by *β* for positive *x* (i.e., 0<*f*(*x*)<*β* for *x*>0), two “anchor” leaves $u,v\in \mathcal {L}$, and a tree *T* equivalent to distance matrix *D* with the corresponding quartet length function *τ*(*Q*), we define:

1 $$\begin{array}{@{}rcl@{}} D^{\prime}_{uv} [a,b] &=& \left\{ \begin{array}{ll} \beta + \alpha. \tau(\{a,b,u,v\}) & ab.uv \notin \mathcal{Q}^{T} \\ \beta - f(\tau(\{a,b,u,v\})) & ab.uv \in \mathcal{Q}^{T} \end{array}\right.  \end{array} $$

2 $$\begin{array}{@{}rcl@{}} D^{\prime}_{v}[a,b] &=& \sum_{u \in \mathcal{L} - \{a,b,v\}} D^{\prime}_{uv}[a,b] \end{array} $$

3 $$\begin{array}{@{}rcl@{}} D^{\prime}[a,b] &=& \sum_{v \in \mathcal{L} - \{a,b\}} \sum_{u \in \mathcal{L} - \{a,b,v\}} D^{\prime}_{uv}[a,b]  \end{array} $$

4 $$\begin{array}{@{}rcl@{}} D^{\prime\prime} [a,b] &=& \max_{u,v \in \mathcal{L} - \{a,b\}} \max\left(0,\frac{D^{\prime}_{uv}[a,b]-\beta}{\alpha}\right).  \end{array} $$

$D^{\prime }, D^{\prime }_{u}$, and $D^{\prime }_{uv}$ are distance matrices on leaf-sets $\mathcal {L}, \mathcal {L}\{v\}$, and $\mathcal {L}-\{u,v\}$, respectively, and are called “all-pairs anchored”, “single anchored”, and “double anchored”. We say $D^{\prime }_{uv}$ is induced from *D* anchored by *u*,*v*. *D* ^′′^ is called an “all-pairs anchored maximum distance matrix” and is defined on the leaf-set $\mathcal {L}$.

#### **Theorem 1**

Let *D* ^*T*^ be an additive distance matrix. A double anchored distance matrix $D^{\prime }_{uv}$ induced from *D* ^*T*^ anchored by arbitrary leaves $u,v\in \mathcal {L}$ is an additive distance matrix for the leaf-set $\mathcal {L}^{\prime }=\mathcal {L}-\{u,v\}$ and corresponds to a tree that is topologically identical to $T|\mathcal {L}^{\prime }$. Similarly, a single anchored distance matrix $D^{\prime }_{v}$ induced from *D* ^*T*^ anchored by an arbitrary leaf and an all-pairs anchored distance matrix *D* ^′^ induced from *D* ^*T*^ are additive distance matrices for the leaf-sets $\mathcal {L}-\{v\}$ and $\mathcal {L}$, respectively, and correspond to trees that are topologically identical to $T|\mathcal {L}-\{v\}$ and *T*, respectively.

#### **Theorem 2**

An All-pairs anchored maximum distance matrix *D* ^′′^ induced from additive matrix *D* ^*T*^ is additive and corresponds to a tree with the identical topology and internal branch lengths to *T*.

Both theorems are proved in the appendix. Theorem 2 is similar to a result given by Brodal et al. [38], and is easy to prove. The basic idea is that for any two non-sister leaves {*a*,*b*}, there is a pair of anchors such that in the resulting quartet, *a* and *b* are not sisters, and the quartet length is exactly the same as the distance between the two leaves minus their terminal branches. We note that similar to us, Brodal et al. use the concept of anchors, but instead of using anchors to *define* distances, they use anchors to efficiently build Buneman trees from *given* distances. Thus, despite some parallels, our anchoring mechanism is novel; In particular, Brodal et al. do not prove our surprising result that a *single arbitrarily chosen* pair of anchors gives additive distances for the correct topology.

Theorem 1 states anchored distances induced from an additive matrix will correspond to the same topology as the initial matrix (albeit with wrong branch lengths). This result is surprising, but its usefulness might be less clear. Theorem 1 enables new estimators of the tree topology that rely on quartets to compute pairwise distances. Let $\mathcal {D}$ denote the input data to be used for inferring a phylogeny. Regardless of the nature of $\mathcal {D}$, we require having a quartet estimator. A quartet estimator is a function that given a quartet of leaves *Q*, uses $\mathcal {D}$ to estimate the quartet tree topology and the quartet length *τ*(*Q*), and is statistically consistent if, as the size of $\mathcal {D}$ increases, the estimated quartet topology and length both converge in probability to correct values. Statistically consistent quartet estimators can be designed for various models (e.g., sequence evolution [39] and the MSC [33, 34]).

Given a statistically consistent quartet estimator, a family of statistically consistent tree inference algorithms can be designed (Additional file 1: Algorithm S1). Details and proofs are given in the (Additional file 1: Section 2.4). The basic idea is the following. We can use the quartet estimator to infer a distance matrix that asymptotically can be made arbitrarily close to an additive distance matrix for the true tree topology. Using a method such as neighbor-joining that infers the correct tree for additive distance matrices with a safety radius will give a consistent estimator of the tree [40].

### DISTIQUE (theory)

**Problem statement:** Given an input dataset $\mathcal {G}$ of a collection of *k* unrooted gene trees, we seek to find the unrooted species tree topology, assuming gene trees are generated by the MSC model [2].

Next, we first describe anchored distances based on the MSC model used in DISTIQUE. We then describe the algorithmic design of DISTIQUE, including its strategies for selecting anchors, combining results from multiple anchors, and dealing with long branches.

#### **Definition 2**

Let *p*(*a* *b*.*u* *v*)denote the true probability of observing the quartet topology *a* *b*.*u* *v* in gene trees generated according to the MSC model. We define MSC-based double, single, and all-pairs anchored distance matrices $D^{*}_{u,v}, D^{*}_{v}$, and *D* ^∗^, respectively on leaf-sets $\mathcal {L}-\{u,v\}, \mathcal {L}-\{v\}$ and $\mathcal {L}$as:

5 $$\begin{array}{@{}rcl@{}} D^{*}_{u,v} [a,b] &=& -\ln p(ab.uv) \end{array} $$

6 $$\begin{array}{@{}rcl@{}} D^{*}_{v}[a,b] &=& \sum_{u \in \mathcal{L} - \{a,b,v\}} -\ln p(ab.uv) \end{array} $$

7 $$\begin{array}{@{}rcl@{}} D^{*}[a,b] &=& \sum_{v \in \mathcal{L} - \{a,b\}} \sum_{u \in \mathcal{L} - \{a,b,v\}} -\ln p(ab.uv) \end{array} $$

#### **Lemma 1**

For species tree estimation under the MSC model, Eq. (1) simplifies to Eq. (5) for *β*= ln3,*α*=1, and *f*(*x*)= ln(3−2*e* ^−*x*^). Thus $D^{\prime }_{uv}[a,b] = D^{*}_{uv} [a,b] =-\ln p(ab.uv).$

#### **Theorem 3**

Given true quartet probabilities $p(ab.uv), D^{*}_{uv}, D^{*}_{v}$, and *D* ^∗^ become additive distance matrices that correspond to the true species tree topology on leaf-sets $\mathcal {L}-\{u,v\}, \mathcal {L}-\{v\}$, and $\mathcal {L}$, respectively.

Lemma 1 is proved in the appendix. From Lemma 1, it follows that Eq. (5) is a special case of Eq. (1); Theorem 3 follows directly from Theorem 1.

It may be surprising that $D^{*}_{uv}$, which is a special case of $D^{\prime }_{uv}$, depends only on quartet topologies and not branch lengths. To see why, readers should recall that *p* is the quart frequency in *gene trees*, and relates to both the quartet topology and the quartet length in the *species tree*.

True quartet probabilities are not known. Instead, we empirically use $\overline {p}(ab.uv)=\frac {1}{k}|\{t:\mathcal {G}|ab.uv\in \mathcal {Q}^{t}\}|$. Empirical frequencies inferred from gene trees converge in probability to true values as the number of genes increases; thus, it is easy to show (proof omitted):

#### **Corollary 1**

$D^{*}_{uv}, D^{*}_{v}$, and *D* ^∗^ computed using empirical frequencies in a random sample of error-free gene trees converge in probability to an arbitrarily small radius of an additive matrix identical in topology to the true species tree; a consistent distance method with a safety radius *[* 40 *]* run on these matrices is a consistent estimator of the species tree topology.

Computing anchored matrices require *Θ*(*n* ^2^ *k*),*Θ*(*n* ^3^ *k*), and *Θ*(*n* ^4^ *k*) time, respectively for $D^{*}_{uv}, D^{*}_{v}$, and *D* ^∗^. Among these matrices, only *D* ^∗^ includes all species.

### DISTIQUE (algorithmic design)

DISTIQUE uses double anchored matrices, which can be each computed in *Θ*(*n* ^2^ *k*). It uses multiple anchors and combines the trees or matrices produced by different anchors. A careful selection of anchors can ensure the final DISTIQUE tree includes all species, and can control its running time between *Θ*(*n* ^2^ *k*) and *Θ*(*n* ^4^ *k*). Before presenting our anchoring strategy, we first need to show how DISTIQUE deals with long branches.

#### Long branches: smoothing and consensus

**Smoothing** For species tree branches that are even moderately long, expected frequencies of alternative quartet topologies become exceedingly close to zero. For example, a species tree quartet length of 12 in coalescent units [41] results in a 99.6 % chance of observing no discordance among 1000 genes. Thus, our simple empirical frequency estimator $\overline {p}$ can easily be equal to zero, resulting in distances of infinity (Eq. 5). To avoid this problem, we use *Krichevsky-Trofimov* [42] (i.e., add-half estimator), which adds a pseudo-count of 0.5 for each of three possible quartet topologies. This estimator has been shown to reach the min-max cumulative loss for KL divergence asymptotically [42].

**Consensus** Smoothing does not fix the larger problem of *distinguishing* between long distances. For example, branches of length 12, 24, or 48 are all very likely to result in no gene tree discordance given 1000 genes; thus, even with smoothing, it remains impossible to distinguish between branches with these very different distances. This limitation makes it impossible to compute distances that reflect the true topology from limited data when the species tree includes adjacent long branches (resembling the saturation problem in phylogenetics [43]). We can construct examples when all gene trees are likely identical, yet our smoothed distances are misleading (Additional file 1: Section 2.2; Figure S7). However, long branches are easy to recover because they appear in most gene trees. A simple majority rule (50 %) consensus of gene trees would return all long branches. Thus, we simply compute the majority consensus and resolve its polytomies using DISTIQUE (Additional file 1: Algorithms S2 and S3). Because the majority consensus is proved *not* positively misleading under the MSC [44], our method remains statistically consistent.

To resolve a polytomy, Additional file 1: Algorithm S2 first assigns a cluster label to each branch pendant to it, and then builds a tree using DISTIQUE with the cluster labels as leaves; this tree defines a resolution of the polytomy. Given anchor species *u*,*v* from two *distinct* clusters, we compute distances between *other* pairs of clusters *A* and *B* using Eq. (5), defining the quartet frequencies as: $\overline {p}(uv.AB) = \frac {1}{|A||B|}\sum _{a\in A}\sum _{b\in B}{\overline {p}(uv.ab)}.$ When all clusters in the consensus tree are correct (expected asymptotically), *p*(*u* *v*.*a* *b*) values are identical; thus, all $\overline {p}(uv.ab)$ values are empirical estimates of the same true value, and using their average is justified.

#### Choosing anchors

Additional file 1: Algorithm S4 shows DISTIQUE’s targeted sampling strategy for choosing a subset of all possible anchor pairs. Let *d*_1_…*d*_*r*_ denote the degree of polytomies in the consensus tree, indexed arbitrarily. For each polytomy *i*, we randomly partition its *d*_*i*_ clusters into sets of size two; if *d*_*i*_ is odd, we randomly choose a cluster and pair it with the remaining cluster. Then, we randomly choose one species from each cluster. This produces $\lceil \frac {d_{i}}{2}\rceil $ pairs of anchors for each polytomy *i*. The total number of anchors is $m={\sum _{1}^{r}} \lceil \frac {d_{i}}{2}\rceil =O(n)$ (Additional file 1: Lemma S2). Each anchor pair is used to resolve all polytomies on the path between them in the consensus tree. This processes may be repeated several rounds (a user-specified input parameter).

Polytomies of degree 4 or 5 cannot be handled using the double anchored approach because once two clusters are chosen as anchors, only two or three clusters remain which cannot be resolved as unrooted trees. For these small polytomies, we always use all-pairs distance matrices; thus, we choose all ${4 \choose 2}$ or ${5 \choose 2}$ possible pairs of clusters around the polytomy. We need *O*(*n*) anchors in this scenario as well (Additional file 1: Lemma S2).

#### Combining anchors

Once *m* anchor pairs are selected, DISTIQUE computes *m* double-anchored matrices and then combines them using one of two methods.

##### Tree-sum:

We first compute *m* trees, each on *n*−2 leaves using the double anchored method (Corollary 1) and then combine these *m* trees using a supertree method. Using a *compatibility supertree* (i.e., one that given a set of compatible input trees, outputs a tree that refines all input trees) would make the approach statistically consistent (Theorem S2, Additional file 1).

We also use the following approach to filter out outlier anchors. We compute an initial supertree from *m* anchored trees, then find the RF distance between *m* trees and the supertree, remove those with an RF distance at least two standard deviations larger than the mean, and recompute the supertree.

##### Distance-sum:

The distance-sum approach creates a summary distance matrix and runs neighbor joining on the summary matrix. The summary distance is simply the average distance of each pair in the set of *m* double anchored matrices. Note that some of the *m* double anchored matrices might not have a value for a given pair of leaves; we treat those as missing values and ignore them when averaging values. The presence of missing values jeopardizes our proofs of statistical consistency.

Let $D^{*}_{uv}$ and $D^{*}_{wz}$ be two double anchored matrices produced using two disjoint pairs of anchors. If the two matrices are reduced to the *n*−4 leaves common between them (i.e., $\mathcal {L}^{\prime }=\mathcal {L}-\{u,v, w,z\}$), we get two matrices that asymptotically converge to an additive matrix for the same tree topology (Corollary 1). The sum of two additive distance matrices that correspond to the same tree topology is also additive for the same topology. Thus, $D^{*}_{uv}|\mathcal {L}^{\prime }+D^{*}_{wz}|\mathcal {L}^{\prime }$ is asymptotically additive for the correct species tree. This provides a theoretical justification for our distance-sum approach. However, distances between four anchors and other leaves are missing in one of the matrices, and thus, their correct placement cannot be guaranteed.

If all ${n \choose 2}$ anchors are used, the distance-sum approach becomes equivalent to the all-pairs approach and is provably statistically consistent (Theorem 3). On the other hand, using only two pairs of anchors makes the placement of anchors dependent on averages of two numbers, one of which is missing, a clearly problematic scenario. Choosing an intermediate number of anchors, while insufficient for giving proofs of consistency, clearly reduces the impact of missing values. For example, assume we have *m* anchors and each species is included in at most only one of those anchors. The summary distance between each pair of leaves becomes an average of *m* values, among which at most one may be missing.

For large enough *m*, we conjecture that the impact of that single missing value is negligible. In the results section, we provide empirical evidence for this conjecture, but future work should explore theoretical proofs. Due to its superior empirical performance, distance-sum is used by default in the DISTIQUE (see Additional file 1: Algorithm S2 for all details).

#### Running time analysis:

Using all-pairs or all-pairs-max clearly require *Θ*(*n* ^4^ *k*) time to build the distance matrix and using the default *O*(*n* ^3^) neighbor joining algorithm [45] would result in *Θ*(*n* ^4^ *k*) total running time. The running times of tree-sum and distance-sum depend on the selection of anchors, and also the exact distance method and supertree method used. Building each double anchored distance matrix requires *Θ*(*n* ^2^ *k*); thus, building *m* matrices requires *Θ*(*n* ^2^ *m* *k*). Using a fast neighbor joining algorithm (e.g., FNJ [46], or NINJA [18]), the running time of distance method can be *O*(*n* ^2^).

Clearly, any function between *Θ*(*n* ^2^ *k*) and *Θ*(*n* ^4^ *k*) can be obtained by adjusting *m*. DISTIQUE’s default strategy requires *O*(*n*) anchors and therefore results in *O*(*n* ^3^ *k*) total running time. For the tree-sum approach, the running time of the supertree method needs to be also added. MRL, which we use here, doesn’t have running time guarantees, but ML methods tend to have average running time close to *O*(*n* ^2^) [47].

### Experimental setup

We use simulated and real datasets to evaluate the accuracy and scalability of DISTIQUE. We measure species tree accuracy using False Negative (FN) rate, which is equivalent to normalized RF distance [48] here because all estimated species trees are fully resolved.

#### Datasets

For biological analyses, we re-analyzed a dataset of 2022 supergene trees from an avian dataset [7 *,* 11]. We also use three sets of simulated datasets we used before: a 37-taxon mammalian dataset [12], a 45-taxon avian dataset [11], and datasets used for evaluating ASTRAL-II [4]. The first two datasets are based on biological data and have a single species tree topology, whereas the last dataset is simulated using SimPhy [49] and has a different species tree per replicate and has heterogeneous parameters. Avian and mammalian datasets enable us to evaluate performance for relatively small numbers of species, varying ILS and the number of genes. The amount of ILS is changed by multiplying or dividing branch lengths by 2 or 5; shorter branches (0.2X and 0.5X) produce more ILS and longer branches reduce ILS (Additional file 1: Table S1). We create two collections for these datasets, one where we fix the number of genes (200 for mammalian and 1000 for avian) and vary the amount of ILS, and a second collection, where we fix the amount of ILS (to very high or 0.2X for mammalian and default 1X for avian) and vary the number of genes (200 to 3200 for mammalian and 200 to 2000 for avian). The simPhy dataset [4] has two collections, and is simulated to capture the range of reasonable biological datasets. In the simPhy-ILS collection, we fix the number of species to 201 and show three levels of ILS, ranging from moderate (10 million generations) to very high (500K generations), and for each case, we vary the number of genes (50, 200, 1000). For each case, we have 100 replicates, half with a speciation rate of 10^−6^ and the other half with 10^−7^. In the simPhy-size, we fix ILS to moderate and speciation rate to 10^−6^, and change the number of species from 10 to 500, with 50 replicates per dataset.

#### Methods

We compare various versions of DISTIQUE, described below, against each other, and against ASTRAL-II [4], which is a quartet-based method, the ASTRID [22] (a new implementation of the NJst algorithm [21]), which is a distance-based method, and concatenation using RAxML [50] (CA-ML). ASTRAL and NJst are statistically consistent summary methods and, like DISTIQUE, work on unrooted gene trees and species trees (most other approaches such as MP-EST and STAR need rooted input). Also, these two are among the most accurate summary methods [3 *,* 4 *,* 21 *,* 22 *,* 51].

##### DISTIQUE:

We explore variants of DISTIQUE, changing the distance matrix (comparing all-pairs, all-pairs-max, tree-sum, and distance-sum; see Additional file 1: Algorithm S1), the number of anchoring rounds (2 to 8), and the use of consensus. To compare to other methods, we use the default distance-sum DISTIQUE (Additional file 1: Algorithm S2), with 2 or 8 rounds of anchoring. DISTIQUE is implemented in python and uses the Dendropy library [52] and uses the FastME [53] as its distance method (but we also tested PhyD* [54]).

## Results

### Comparison between DISTIQUE variants

We start by comparing all-pairs and all-pairs-max variants, each applied to either the entire set of species or to polytomies of a 50 % majority rule consensus (default), limiting our study to the 37-taxon and 45-taxon avian and mammalian datasets where *Θ*(*n* ^4^ *k*) methods could run. On both datasets, a surprising pattern emerges. Without the use of consensus, the error unexpectedly goes up with decreased ILS, a pattern that is more pronounced for all-pairs-max (Additional file 1: Figures S1 and S2). As discussed before, we attribute this pattern to difficulties of estimating long quartet lengths. When consensus is used within DISTIQUE, the accuracy improves with decreased ILS, as expected (Additional file 1: Figures S1 and S2). Depending on the level of ILS, the consensus tree is unresolved for 25 to 95 % of branches, leaving much to DISTIQUE to resolve. Overall, all-pairs methods has better accuracy than all-pairs-max, a result that we do not find surprising. Based on these results, hereafter, we only show results for DISTIQUE applied to a majority consensus, and we omit all-pairs-max.

We compared the three algorithms, all-pairs, tree-sum, and distance-sum (the last two with eight rounds of anchor sampling), and observed that the distance-sum is competitive with all-pairs and outperforms tree-sum (Table 1). The difference between all-pairs and distance-sum was never more than 1 %. Distance-sum consistently outperformed tree-sum, by as much as 5 % in some cases, despite the fact that tree-sum is provably consistent and distance-sum has not been proved consistent. Thus, we chose to set the default DISTIQUE implementation to distance-sum.

**Table 1:** DISTIQUE variants on simulated datasets

Dataset	#genes	All-pairs	Tree-sum	Distance-sum
avian-0.5X	1000	**0.10**	0.11	0.11
avian-1X	1000	**0.08**	0.09	**0.08**
avian-2X	1000	**0.05**	0.08	0.06
mammalian-0.2X	200	**0.11**	0.13	**0.11**
mammalian-0.5X	200	**0.06**	0.12	0.07
mammalian-1X	200	**0.04**	0.08	**0.04**
mammalian-2X	200	**0.02**	0.04	**0.02**
simphySize-10	50	0.03	0.03	0.03
simphySize-10	200	0.02	0.02	0.02
simphySize-10	1000	0.02	0.02	0.02
simphySize-50	50	**0.07**	0.10	**0.07**
simphySize-50	200	**0.04**	0.07	**0.04**
simphySize-50	1000	0.03	0.04	0.04
simphySize-100	50	**0.08**	0.11	**0.08**
simphySize-100	200	**0.05**	0.06	**0.05**
simphySize-100	1000	**0.03**	0.05	0.04

Distance-sum and tree-sum are both based on 8 rounds. For simPhy-size, all-pairs could not finish given two days of running time for more than 100 species. Where there is at least 1 % difference between methods, the best method is shown in bold

We next evaluated the impact of anchor sampling by changing the number of rounds of targeted sampling between 1 and 8 on the avian and mammalian datasets (Additional file 1: Figures S3 and S4). The distance-sum method had substantial improvements when going from one to two rounds, and generally much smaller improvements after that. We show results for both 2 and 8 rounds when comparing DISTIQUE to other methods.

Finally, we checked the impact of the exact distance method used inside DISTIQUE (Additional file 1: Figure S5), using a variety of methods implemented inside FastME [53] and PhyD* [54] on both mammalian and avian datasets. There were substantial variations of accuracy among distance methods, especially on the avian dataset. PhyD* tended to have more error, and among methods implemented in FastME, balanced minimum evolution (BME) with SPR moves had the highest accuracy. We chose this option of FastME in the default DISTIQUE.

### DISTIQUE versus other methods

**Fig. 1:**
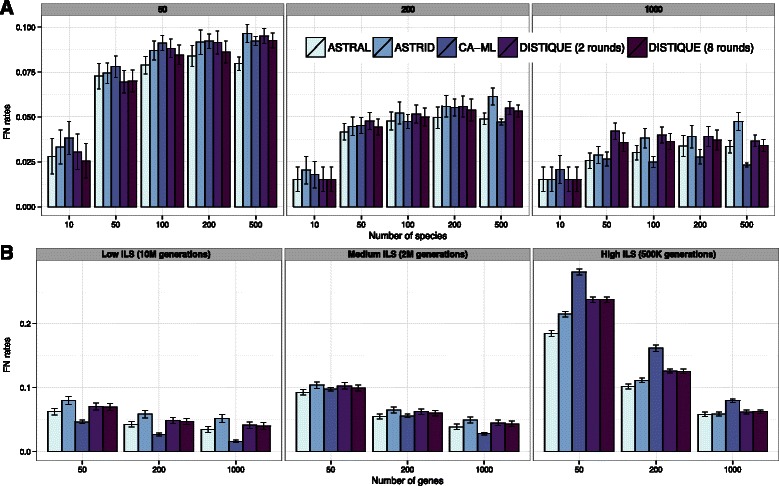
DISTIQUE versus other methods on (**a**) simPhy-size and (**b**) simPhy-ILS datasets using estimated gene trees. *Boxes*: (**a**) number of genes and (**b**) levels of ILS. The mean and standard error of species tree error are shown over (**a**) 50 and (**b**) 100 replicates

**Fig. 2:**
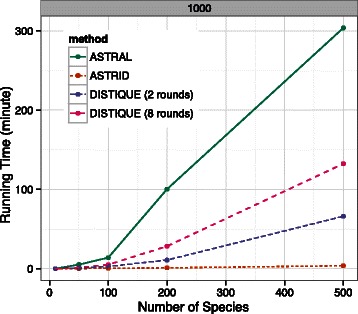
Running time comparisons on the simPhy-size datasets with 1000 genes (Additinal file 1: Figure S6 has other numbers of genes). *Lines* show the average running times (50 replicates) in hours

**simPhy-size:** On this simulated dataset, we compare running time and tree accuracy across methods. Generally, all the methods we studied had similar patterns of accuracy on the simPhy-size dataset, and the mean errors of different methods tended to be within the standard error of each other (Fig. 1 a). According to a two-way ANOVA test with FDR correction [55] for multiple testing (*n*=24; see caption of Additional file 1: Table S3) with *α*=0.05, the error rate of DISTIQUE-8 was statistically indistinguishable from both ASTRAL and ASTRID (Additional file 1: Table S3). In the few cases where the differences seemed substantial, for example on 500 species and 1000 genes, ASTRAL tended to be the best, followed by both versions of DISTIQUE (but there were exceptions; e.g., 50 species and 1000 genes). Unlike the accuracy, running times of summary methods were quite different (Fig. 2). ASTRID was by far the fastest, followed by DISTIQUE-2 and DISTIQUE-8, and ASTRAL was the slowest. For example, with 500 species and 1000 genes, DISTIQUE-2 and DISTIQUE-8 ran in about 1.1 and 2.2 hours, while ASTRAL took 5 hours, and ASTRID took only 7.5 minutes.

**simPhy-ILS:** On the simPhy-ILS dataset where the number of species is fixed to 201, differences between various summary methods were generally small (Fig. 1 b), but overall, ASTRAL was significantly better than DISTIQUE-8 (*p*<0.001). However, DISTIQUE-8 and ASTRID were indistinguishable (Additional file 1: Table S3). The magnitude of the difference between ASTRAL and DISTIQUE-8 significantly depended on the level of ILS (*p*=0.001), where with low or medium ILS levels, the two methods had a similar error, but with increased ILS, ASTRAL outperformed DISTIQUE; the differences were more pronounced when we had fewer gene trees (significant: *p*=0.039; Additional file 1: Table S3).

**Fig. 3:**
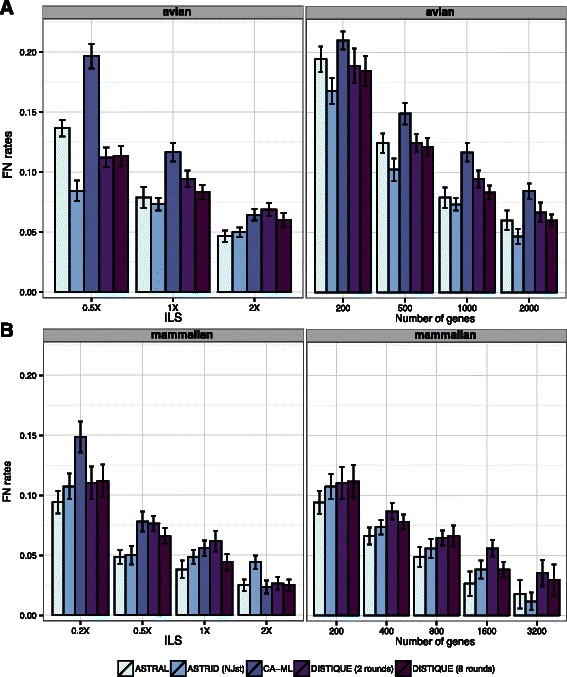
The accuracy of methods on Avian (**a**) and Mammalian (**b**) datasets using estimated gene trees. *Left*: number of genes is fixed (1000 for avian, 200 for mammalian) and ILS levels change. *Right*: ILS level is fixed (default 1X for avian and very high 0.2X for mammalian). We show average and standard error over 20 replicates, except for 1600 and 3200 genes, which have 10 and 5 replicates, respectively. For the mammalian dataset with 0.2X ILS, due to the large number of gene trees, running concatenation was not feasible

**Avian** On the avian dataset (Fig. 3 a), ASTRID was generally the best method, followed by DISTIQUE-8 (which was significantly worse; *p*=0.004) and then ASTRAL; CA-ML was the worst. Differences between ASTRAL and DISTIQUE-8 were not statistically significant (Additional file 1: Table S3). The largest difference between DISTIQUE-8 and the best method was for 0.5X ILS, where DISTIQUE-8 had 2.9 % more error than ASTRID.

**Mammalian** On this dataset (Fig. 3 b), overall, ASTRAL was the best method, and was significantly better than DISTIQUE (*p*=0.025). DISTIQUE and ASTRID were overall statistically indistinguishable (Additional file 1: Table S3). The relative error of concatenation depended on the level of ILS, which was much worse than summary methods for high levels of ILS, but better for low levels of ILS.

### Biological results

On the avian dataset, we ran ASTRAL, ASTRID, and DISTIQUE-8 and used both bootstrapping [56] and local posterior probability (pp) [57] to quantify branch support (Additional file 1: Figures S8 and S9). Bootstrap support was generally high, but the local pp was low for many branches. DISTIQUE and ASTRID differed on three branches. Of these, one, related to the first neoavan split, had high local pp support in ASTRID (0.98) but very low local pp in DISTIQUE; the remaining conflicts had local pp below 0.58 in both trees. ASTRAL and DISTIQUE differed in six branches, and all of these had local pp below 0.58 in DISTIQUE, and all but one also had low local pp (≤ 0.9) in ASTRAL. None of these conflicting relationships have been well resolved in the literature. Interestingly, many of conflicting branches with low local pp had high bootstrap support. It can be argued that conflicts are due to uncertainties resulting from insufficient data, but bootstrapping misleadingly computes high support [57].

## Discussion

We compared three statistically consistent summary methods, ASTRAL, ASTRID, and DISTIQUE; overall, ASTRAL was at least as good as other methods on most datasets, but ASTRID was occasionally the best. DISTIQUE was often as good as and never more than 3 % worse than the best method. The choice of the best method depended on the level of ILS and the number of genes, suggesting when the level of ILS is expected to be very high, ASTRAL might be the best choice. On the other hand, the running time of DISTIQUE grows more slowly with increased numbers of genes; for datasets with large number of species and tens of thousands of genes, DISTIQUE and ASTRID provide fast alternatives to ASTRAL.

Despite having strong competition in ASTRAL and ASTRID, we believe DISTIQUE is a promising approach, for several reasons. Because of its speed, DISTIQUE can be used for a very fast estimation of species trees, for example, as a starting point for an extensive hill-climbing search. DISTIQUE can also generate a set of trees instead of a single tree, and we plan to study whether these sets of trees can be utilized for defining the search space of ASTRAL.

DISTIQUE is essentially a method for 1) defining distances based on quartets, and 2) subsampling the space of all *Θ*(*n* ^4^) quartets. The first aspect of DISTIQUE can be replaced by improved ways of defining distances, for example those that better handle gene tree estimation error. Co-estimation of gene trees and the species tree [58] is a computationally challenging problem in general. However, it is reasonable to think that a similar problem defined on quartets, and addressed using distances becomes easier, as some recent theoretical results suggest [32 *,* 59]. DISTIQUE provides a general way for using anchoring introduced in this paper to implement novel distance-based gene tree species tree co-estimation in a scalable fashion. Simpler approaches of taking into account gene tree uncertainty, for example weighting various quartets according to coalescent expectations, might also result in improvements. Finally, we note that DISTIQUE’s anchoring strategy can be paired with site-based ILS methods such as SVDQuartets [35], and more broadly for other tree inference problems.

## Conclusions

We introduced a general approach for computing tree leaf distances by inferring topologies and internal branch lengths for quartets of leaves. We used our novel anchoring to design DISTIQUE, a new statistically consistent summary method for species tree estimation. DISTIQUE has variants, with several strategies for choosing and combining anchors. The default version of DISTIQUE requires *O*(*n* ^3^ *k*) running time and is much faster than ASTRAL. In terms of accuracy, DISTIQUE was nearly as accurate as ASTRAL with differences that were rarely substantial.

**Fig. 4:**
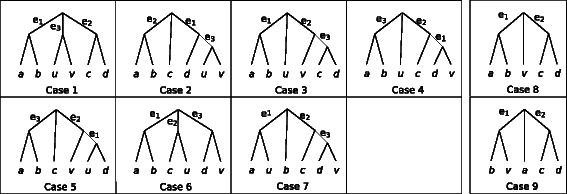
Possible ways of adding anchors to a quartet. *Left*: All 7 possible placements of two anchors *u* and *v* on a given quartet topology *a* *b*.*c* *d*. Internal branches are labeled with their length. *Right*: Placements of a single anchor *v* on quartet tree *a* *b*.*c* *d*

## Appendix

### Proof of theorems

#### *Proof of Theorem *1

Let $\{a,b,c,d\}\subset \mathcal {L}$ be four arbitrary leaves and $\mathcal {L}^{\prime }=\mathcal {L}- \{a,b,c,d\}$. W.l.o.g assume $ab.cd \in \mathcal {Q}^{T}$. We prove that the four point conditions hold for this arbitrarily chosen quartet in $D^{\prime }_{uv},D^{\prime }_{v},$ and *D* ^′^; we also prove that the four point conditions are true for a tree compatible with the tree *T*. Proving these conditions for arbitrary quartets completes the proof by results of Buneman [37].

We start with the double-anchored matrix. The four point condition can be written in three ways, but only one of them is compatible with the tree *T*. Since we assumed w.l.o.g that $ab.cd \in \mathcal {Q}^{T}$, the four point condition compatible with *T* is:

$$\begin{array}{*{20}l} \overbrace{D^{\prime}_{uv}[a,b]+D^{\prime}_{uv}[c,d]}^{L} &< \overbrace{D^{\prime}_{uv}[a,d] + D^{\prime}_{uv}[b,c]}^{R1} \\ &= \overbrace{D^{\prime}_{uv}[a,c] + D^{\prime}_{uv}[b,d]}^{R2}. \end{array} $$

Figure 4 shows all ways of placing anchors {*u*,*v*} on the quartet tree *a* *b*.*c* *d*. Anchors can be sisters, placed on the internal branch (Case 1) or on a tip branch (Case 2; w.l.o.g, we pick the branch pending to *d*). When anchors are not sisters, they can be both placed on the internal branch (Case 3), or one on the internal branch and the other on a tip branch (Case 4), or they can be both on terminal branches, which can be done in three ways: *u* and *v* can be on the same terminal branch (Case 5), on different but adjacent branches (Case 6), or on non-adjacent branches (Case 7).

In Table 2, for each of the seven cases, we compute *L*,*R*1,*R*2. We use Eq. (1) to derive $D^{\prime }_{uv}[x,y]$ values. Where *x* *y*.*u* *v* is induced by the tree shown in Fig. 4, we use [*β*−*f*(*t*)] and otherwise we use [*β*+*α* *t*], where *t*=*τ*(*x*,*y*,*u*,*v*) is the length of the internal branch for the quartet tree induced by {*x*,*y*,*u*,*v*}. For example, for Case 1, $D^{\prime }_{uv}[a,b]=[\beta -f(e_{1}+e_{3})]$ because *a* *b*.*u* *v* is induced by the tree, and the length of the edge on the *a* *b*.*u* *v* quartet tree is *e*_1_+*e*_3_; in Case 7, $D^{\prime }_{uv}[a,b]=\beta +\alpha e_{1}$ because *a* *b*.*u* *v* is *not* induced by the tree and *τ*(*a*,*b*,*u*,*v*)=*e*_1_.

We need to show that *L*<*R*1 and *R*1=*R*2. We remind the reader that all branches are assumed to be strictly positive and that *f* is a positive and monotonically increasing function bounded from above by *β*. In all cases, the equality of *R*1 and *R*2 is immediately clear from the Table 2. The inequality *L*<*R*1 follows directly from the fact that *f*(*x*) is monotonically increasing in Cases 1, 2, and 5. For Case 3, because of positivity of *f*(*x*) and *α*, we have *L*<2*β*<*R*. Similarly, for Case 4, *L*<2*β*+*α* *e*_1_<2*β*+*α* *e*_1_+2*α* *e*_2_=*R*. Case 6 follows from the positivity of *f*, and Case 7 is trivially correct for positive branch lengths. Thus, we have shown in all possible relationships between {*u*,*v*} and the quartet tree, the four point condition holds for the topology consistent with tree *T*. Therefore, the proof is complete for the double anchored case.

**Table 2:** Proof of four-point condition for double anchors. Four point condition for all 7 cases of adding {*u*,*v*} to a quartet tree, as shown in Fig. 4 (left side)

	$L =D^{\prime }_{uv}[a,b]+D^{\prime }_{uv}[c,d]$	$ R1 =D^{\prime }_{uv}[a,d] + D^{\prime }_{uv}[b,c]$	$R2 =D^{\prime }_{uv}[a,c] + D^{\prime }_{uv}[b,d]$
Case 1	[*β*−*f*(*e*_1_+*e*_3_)]+[*β*−*f*(*e*_2_+*e*_3_)]	[*β*−*f*(*e*_3_)]+[*β*−*f*(*e*_3_)]	[*β*−*f*(*e*_3_)]+[*β*−*f*(*e*_3_)]
Case 2	[*β*−*f*(*e*_1_+*e*_2_+*e*_3_)]+[*β*−*f*(*e*_3_)]	[*β*−*f*(*e*_3_)]+[*β*−*f*(*e*_1_+*e*_3_)]	[*β*−*f*(*e*_1_+*e*_3_)]+[*β*−*f*(*e*_3_)]
Case 3	[*β*−*f*(*e*_1_)]+[*β*−*f*(*e*_3_)]	[*β*+*α* *e*_2_]+[*β*+*α* *e*_2_]	[*β*+*α* *e*_2_]+[*β*+*α* *e*_2_]
Case 4	[*β*−*f*(*e*_3_)]+[*β*+*α* *e*_1_]	[*β*+*α*(*e*_1_+*e*_2_)]+[*β*+*α* *e*_2_]	[*β*+*α* *e*_2_]+[*β*+*α*(*e*_1_+*e*_2_)]
Case 5	[*β*−*f*(*e*_2_+*e*_3_)]+[*β*+*α* *e*_1_]	[*β*−*f*(*e*_2_)]+[*β*+*α* *e*_1_]	[*β*−*f*(*e*_2_)]+[*β*+*α* *e*_1_]
Case 6	[*β*−*f*(*e*_1_)]+[*β*+*α*(*e*_2_+*e*_3_)]	[*β*+*α* *e*_3_]+[*β*+*α* *e*_2_]	[*β*+*α* *e*_2_]+[*β*+*α* *e*_3_]
Case 7	[*β*+*α* *e*_1_]+[*β*+*α* *e*_3_]	[*β*+*α*(*e*_1_+*e*_2_+*e*_3_)]+[*β*+*α* *e*_2_]	[*β*+*α*(*e*_1_+*e*_2_)]+[*β*+*α*(*e*_2_+*e*_3_)]

Now consider the “single anchored” distance matrix $D^{*}_{v}$ on the leaf-set $\mathcal {L}-\{v\}$ (for a single $v\in \mathcal {L}$). To prove additivity of the single anchored distance matrix, we need to prove the following four point condition:

$$\begin{array}{@{}rcl@{}} & \sum_{u \notin \{a,b\}} D^{\prime}_{uv}[a,b] + \sum_{u \notin \{c,d\}} D^{\prime}_{uv}[c,d] &< \\ & \sum_{u \notin \{a,d\}} D^{\prime}_{uv}[a,d] + \sum_{u \notin \{a,b\}} D^{\prime}_{uv}[b,c] &= \\ & \sum_{u \notin \{a,c\}} D^{\prime}_{uv}[a,c] + \sum_{u \notin \{b,d\}} D^{\prime}_{uv}[b,d] \end{array} $$

We divide each sum to terms with $u \in \mathcal {L}^{\prime }$ and $u \notin \mathcal {L}^{\prime }$:

$$\begin{array}{*{20}l} &\sum\limits_{u \in \mathcal{L}^{\prime}} D^{\prime}_{uv}[a,b] + D^{\prime}_{uv}[c,d] + \\ &\underbrace{ D^{\prime}_{cv}[a,b] + D^{\prime}_{dv}[a,b] + D^{\prime}_{av}[c,d] + D^{\prime}_{bv}[c,d]}_{L} < \\ &\sum\limits_{u \in \mathcal{L}^{\prime}} D^{\prime}_{uv}[a,d] + D^{\prime}_{uv}[b,c] +\\ &\underbrace{D^{\prime}_{bv}[a,d] + D^{\prime}_{cv}[a,d] + D^{\prime}_{av}[b,c] + D^{\prime}_{dv}[b,c]}_{R1} =\\ &\sum\limits_{u \in \mathcal{L}^{\prime}} D^{\prime}_{uv}[a,c] + D^{\prime}_{uv}[b,d] +\\ &\underbrace{D^{\prime}_{bv}[a,c] + D^{\prime}_{dv}[a,c] + D^{\prime}_{av}[b,d] + D^{\prime}_{cv}[b,d]}_{R2} \end{array} $$

For $u \in \mathcal {L}^{\prime }$ terms, the sums are exactly those we analyzed for double anchored distances; thus, the additivity is already proved. Since the sum of two additive distances is additive, it suffices to prove additivity for *u*∈{*a*,*b*,*c*,*d*} cases, marked as *L*,*R*1, and *R*2 above.

A single anchor *v* can be placed (Fig. 4) either on the internal branch (Case 8) or on a terminal branch (Case 9) of a quartet tree. We prove *L*<*R*1=*R*2 for these: In Case 8, we have:

$$\begin{array}{*{20}l} {}L = & [\beta -f(e_{1})] \!+ [\beta -f(e_{1})]+[\beta -f(e_{2})]+ [\beta -f(e_{2})] \\ < & 4\beta <[\beta +\alpha e_{1}]+[\beta +\alpha e_{1}] + [\beta +\alpha e_{2}] = R1=R2 \end{array} $$

and in case 9,

$${{}\begin{aligned} L & = 2 [\beta +\alpha e_{1}] + [\beta - f(e_{2})] +[\beta -f(e_{1}+e_{2})] <\\ &\quad 4\beta+2\alpha e_{1} - f(e_{1}+e_{2}) <\\ &\quad [\beta \,+\, \alpha e_{2}] \,+\, [ \beta -f(e_{1}) ] +[\beta + \alpha e_{1}] + [\beta +\alpha (e_{1}+e_{2})]\\ & = R1=R2. \end{aligned}} $$

Note that the four point condition proved above is for the topology that corresponds to the tree *T*. The proof for single-anchored distances follows from additivity of sum of additive matrices.

We now prove the additivity for the all-pairs matrix. Equation (3) has three types of terms: {*u*,*v*}∩{*a*,*b*,*c*,*d*} may have (I) both anchors, (II) one anchor, or (III) none. The four point condition can be written:

$${{}\begin{aligned} \overbrace{2 D^{\prime}_{ab}[c,d]}^{I} + &\overbrace{\sum\limits_{v \in \mathcal{L}^{\prime}} \sum\limits_{u \in \{c,d\}} D^{\prime}_{uv}[a,b] + \sum\limits_{u \in \{a,b\}}D^{\prime}_{uv}[c,d]}^{II} + & \\ &\overbrace{\sum\limits_{u,v \in \mathcal{L}^{\prime}} D^{\prime}_{uv}[a,b] + D^{\prime}_{uv}[c,d]}^{III} &< \\ 2 D^{\prime}_{ad}[b,c] +& \sum\limits_{v \in \mathcal{L}^{\prime}} \sum\limits_{u \in \{b,c\}} D^{\prime}_{uv}[a,d] +\sum\limits_{u \in \{a,d\}} D^{\prime}_{uv}[b,c] +&\\ &\sum\limits_{u,v \in \mathcal{L}^{\prime}} D^{\prime}_{uv}[a,d] + D^{\prime}_{uv}[b,c] &=\\ 2 D^{\prime}_{ac}[b,d] + &\sum\limits_{v \in \mathcal{L}^{\prime}} \sum\limits_{u \in \{b,d\}} D^{\prime}_{uv}[a,c] +\sum\limits_{u \in \{a,c\}} D^{\prime}_{uv}[b,d] +&\\ &\sum\limits_{u,v \in \mathcal{L}^{\prime}} D^{\prime}_{uv}[a,c] + D^{\prime}_{uv}[b,d] \end{aligned}} $$

For terms of type (III) and (II), the additivity is already proved in double and single anchored cases, respectively. Thus, we need to prove additivity only for terms of type (I), which have no anchors. Let *x*=*τ*(*a*,*b*,*c*,*d*).

$$\begin{array}{*{20}l} &2D^{\prime}_{ab}[c,d] = 2[\beta-f(x)]< 2\beta<2[\beta+\alpha x] = \\ & 2D^{\prime}_{ad}[b,c] = 2D^{\prime}_{ac}[b,d] \end{array} $$

Thus, for all three types, the four point conditions hold for the topology found in *T*. Proof follows from the fact that the sum of additive terms is additive. □

#### *Proof of Theorem *2

We prove that Eq. (4) returns the sum of internal branch lengths on the path from *a* to *b* on the tree *T* (we denote this by *D* *T*_*ab*_). The theorem immediately follows because a distance matrix compatible with the tree *T* has to be by definition additive and compatible with it (note that the theorem also claims that *D* ^′′^ gives internal branch lengths). For simplicity, we prove with *α*=1; extension to other values is simple. If *a* and *b* are not sisters in *T*, there exists an anchor pair (*u*,*v*) with quartet topology *a* *u*.*b* *v* and $\tau (a,b,u,v)=D^{T}_{ab}$; to find such *u* and *v*, the following procedure can be followed. Pick *u* arbitrarily from the sister group of *a* after rooting *T* on *b* and pick *v* arbitrarily from the sister group of *b* after rooting *T* on *a*. With this choice, it’s easy to see that *τ*(*a*,*b*,*u*,*v*) becomes simply the sum of internal branches between *a* and *b*; thus, from the first case of Eq. (1), we have $D^{\prime }_{uv}[a,b]-\beta =\frac {\tau (a,b,u,v)}{\alpha }+\beta -\beta =D^{T}_{ab}$. Moreover, $D^{\prime }_{wz}[a,b]-\beta $ for two other anchors *w*,*z* cannot be bigger than $D^{T}_{ab}$. That is because if $ab.wz\in \mathcal {Q}^{T}$, then $D^{\prime }_{wz}[a,b]<\beta $; else, *τ*(*a*,*b*,*w*,*z*) will give the length for a subpath from *a* to *b*. Thus, the max function in Eq. (4) returns $D^{T}_{ab}$, as desired. When (*a*,*b*) are sisters, $D^{T}_{ab}=0$; also $D^{\prime }_{uv}[a,b]<0$ for any (*u*,*v*), and thus, the max function returns *D* ^′′^[*a*,*b*]=0; this is what we want, since for sisters, the length of the internal branch length is zero. Thus, as desired, Eq. (4) always returns the length of the internal branches in the *T* between *a* and *b*; this completes the proof. □

#### *Proof of Lemma *1

For *x*>0, ln(3−2*e* ^−*x*^) is clearly positive, monotonic, and bounded from above by ln3, as required by Definition 1. Let *Q*={*a*,*b*,*u*,*v*} and let *T* be the true species tree. To prove that Eq. (1) simplifies to (5), consider two cases. If anchors *u*,*v* are not sisters in the species tree quartet on *Q* (i.e., $ab.uv\notin \mathcal {Q}^{T}$), by the MSC model, $p(ab.uv)=\frac {1}{3}e^{-\tau (Q)}$ and thus, *τ*(*Q*)=− ln3*p*(*a* *b*.*u* *v*). In the first case in (1), $D^{\prime }_{uv}=\beta + \alpha. \tau (Q) = \ln 3+ \tau (Q)= \ln 3 - \ln 3p(ab.uv)=- \ln p(ab.uv)$. In the second case, *u*,*v* are sisters (i.e., $ab.uv\in \mathcal {Q}^{T}$), and by the MSC model, $p(au.bv)=1-\frac {2}{3}e^{-\tau (Q)}$; thus, $\tau (Q)=-\ln \frac {3}{2}(1-p(au.bv))$. In the second case in (1), the distance is *β*−*f*(*τ*(*Q*))= ln3− ln(3−2*e* ^−*τ*(*Q*)^)=− ln*p*(*a* *b*.*u* *v*). Thus, in both cases, $D^{\prime }_{uv} = D^{*}_{uv}$. □

## Additional file

Additional file 1 Supplementary.pdf. The Supplementary Material for the paper. (PDF 426 KB)

## Abbreviations

ANOVA: Analysis of variance
ASTRAL: Accurate species tree algorithm
ASTRID: Accurate species trees from Internode distances
CA-ML: Concatenated analysis using maximum likelihood
DISTIQUE: Distance-based inference of species trees from induced quartet elements
FN: False negative
ILS: Incomplete lineage sorting
MSC: Multi species coalescent
RF distance: Robinson-foulds distance
RAxML: Randomized axelerated maximum likelihood
SOM: Supplementary online material
