# Supplementary material for: Anchoring quartet-based phylogenetic distances and applications to species tree reconstruction
Source: BMC Genomics. 2016 Nov 11;17(Suppl 10):783. doi: 10.1186/s12864-016-3098-z (PMC5123309; doi:10.1186/s12864-016-3098-z)
Supplement: Supplementary file 1 — Supplementary.pdf. The Supplementary Material for the paper. (PDF 426 KB) [file 12864_2016_3098_MOESM1_ESM.pdf]

Anchored distances for quartet-based estimation of phylogenetic trees and applications to coalescent-based analyses (Supplementary Matrial)

Erfan Sayyari[1] and Siavash Mirarab*[1]

[1]University of California, San Diego, Department of Electrical and Computer Engineering

Contents

1 Supplementary Figures and Tables **3**
- 1.1 Supplementary Tables . . . 3
- 1.2 Supplementary Figures . . . 5

2 Supplementary theoretical results **13**
- 2.1 Supplementary lemmas . . . 13
- 2.2 Difficulties with long branches and need for majority consensus . . . 14
- 2.3 Computing pseudo-counts for allpairs-max . . . 15
- 2.4 Tree inference algorithms . . . 15

3 Commands and version numbers **21**

List of Figures

- S1 **Accuracy of different implementations of DISTIQUE for the Mammalian dataset.** . . . 6
- S2 **Accuracy of different implementations of DISTIQUE for Avian dataset.** . . . 7
- S3 **Impact of number of rounds of anchor sampling on accuracy.** . . . 8
- S4 **Impact of number of rounds of anchor sampling on accuracy.** . . . 9
- S5 **Impact of distance method on accuracy.** . . . 10
- S6 **Running times of DISTIQUE versus other methods for the simPhy-size dataset.** . . . 10
- S7 **An example where long branches can cause problems.** . . . 11
- S8 **Species trees generated using DISTIQUE on the Avian biological dataset [1].** 12
- S9 **Species trees generated using ASTRID (NJst) and ASTRAL on Avian biological dataset [1].** . . . 12

List of Tables

- S1 **Empirical statistics of simulated Avian and Mammalian datasets.** . . . 3
- S2 **Empirical statistics of simulated 11-taxon dataset [2]** . . . 3
- S3 **Two-way ANOVA test with FDR correction** . . . 4
- S4 **Description of variables in Algorithm S2** . . . 17

*Corresponding author: smirarab@ucsd.edu

List of Algorithms

S1 **Anchored quartet-based algorithms** 16
S2 **DISTIQUE distance sum algorithm** 18
S3 **DISTIQUE distance sum algorithm - continued** 19
S4 **Anchor selection algorithm** 20

1 Supplementary Figures and Tables

1.1 Supplementary Tables

Table S1: **Empirical statistics of simulated Avian and Mammalian datasets.** Model condition $2X$ corresponds to the case where ILS is reduced by increasing the branch lengths (2 times longer), and $0.5X$ represents the case where ILS is increased by reducing the branch lengths (2 times shorter). In the same way, the model condition with $0.2X$ corresponds to the case where ILS is reduced by dividing the branch lengths by five. Average Robinso-Foulds (RF) distances between true gene trees and the model species tree are provided in *AD to species tree*. *# gene trees* shows the number of gene trees that are available for the corresponding dataset and ILS. *#base pairs* represents the number of base pairs, and *# replicates* shows the number of replicates for the corresponding dataset and ILS. In column *Ref.*, the reference paper for each dataset is provided. For the Mammalian with ILS level $0.2X$, *# replicates* 5 and 10 are for the model conditions where *# gene trees* is 3200, and 1600 respectively. Also for the Avian dataset with ILS level $1X$, *# replicates* 10 is only for the model condition with *# gene trees* 2000.

	ILS	AD to species tree	# gene trees	# base pairs	# replicates	Ref.
Mammalian	$2X$	18%	200	500,true	20	[3]
	$1X$	32%	200	500,true	20	[3]
	$0.5X$	54%	200	500,true	20	[3]
	$0.2X$	79%	100, 200, 400, 800, 1600, 3200	500,true	5, 10, 20	[3]
Avian	$2X$	35%	1000	500,true	20	[4]
	$1X$	47%	200, 500, 1000, 2000	500,true	10, 20	[4]
	$0.5X$	59%	1000	500,true	20	[4]

Table S2: **Empirical statistics of simulated 11-taxon dataset [2]**. Model condition M1 corresponds to the very low ILS, model condition M2 corresponds to low ILS, model condition M3 shows high ILS, and model condition M4 for very high ILS. *AD* represents average bipartition distance between true gene trees and true species trees, expressed as a percentage. The rest of columns are the same as Table S1.

dataset	AD	# base pairs	# gene trees	# replicates	Reference
11-taxon M1	15.5%	10, 25, 50, 100, 200	100, 500, 1000	50	[2]
11-taxon M2	38.3%	10, 25, 50, 100, 200	100, 500, 1000	50	[5]
11-taxon M3	66.3%	10, 25, 50, 100, 200	100, 500, 1000	50	[2]
11-taxon M4	85.0%	10, 25, 50, 100, 200	100, 500, 1000	50	[5]

Table S3: **two-way ANOVA test with FDR correction**. A two-way ANOVA test with FDR correction for multiple testing ($n = 24$) with $\alpha = 0.05$ was conducted to investigate statistically significant differences between different methods. Values bellow $\alpha = 0.05$ (statistically significant values) are shown in bold. Note that column *effects of method* shows the p-values of the impact of the choice of methods (DISTIQUE-8 vs method specified in column *method*). The column *method vs ILS or # species* shows p-values of effects of different levels of ILS (for simPhy-ILS, avian, and mammalian) or different number of species (for simPhy-size) on the relative errors of DISTIQUE-8 and the other method being compared (ASTRID or ASTRAL). The last column shows the effects of varying numbers of gene trees on the relative performance of the methods. FDR correction was applied on all p-values shown in the table (thus, $n = 24$).

dataset	method	effects of method	method vs ILS or # species	method vs # gene trees
avian	astral	0.904	0.191	0.991
avian	astrid	**0.004**	0.904	0.991
mammalian	astral	**0.025**	0.904	0.991
mammalian	astrid	0.904	0.226	0.991
simPhy-ILS	astral	$\mathbf{< 1e^{-6}}$	**0.001**	**0.039**
simPhy-ILS	astrid	0.966	**0.001**	0.904
simPhy-size	astral	0.121	0.991	0.991
simPhy-size	astrid	0.137	0.991	0.991

1.2 Supplementary Figures

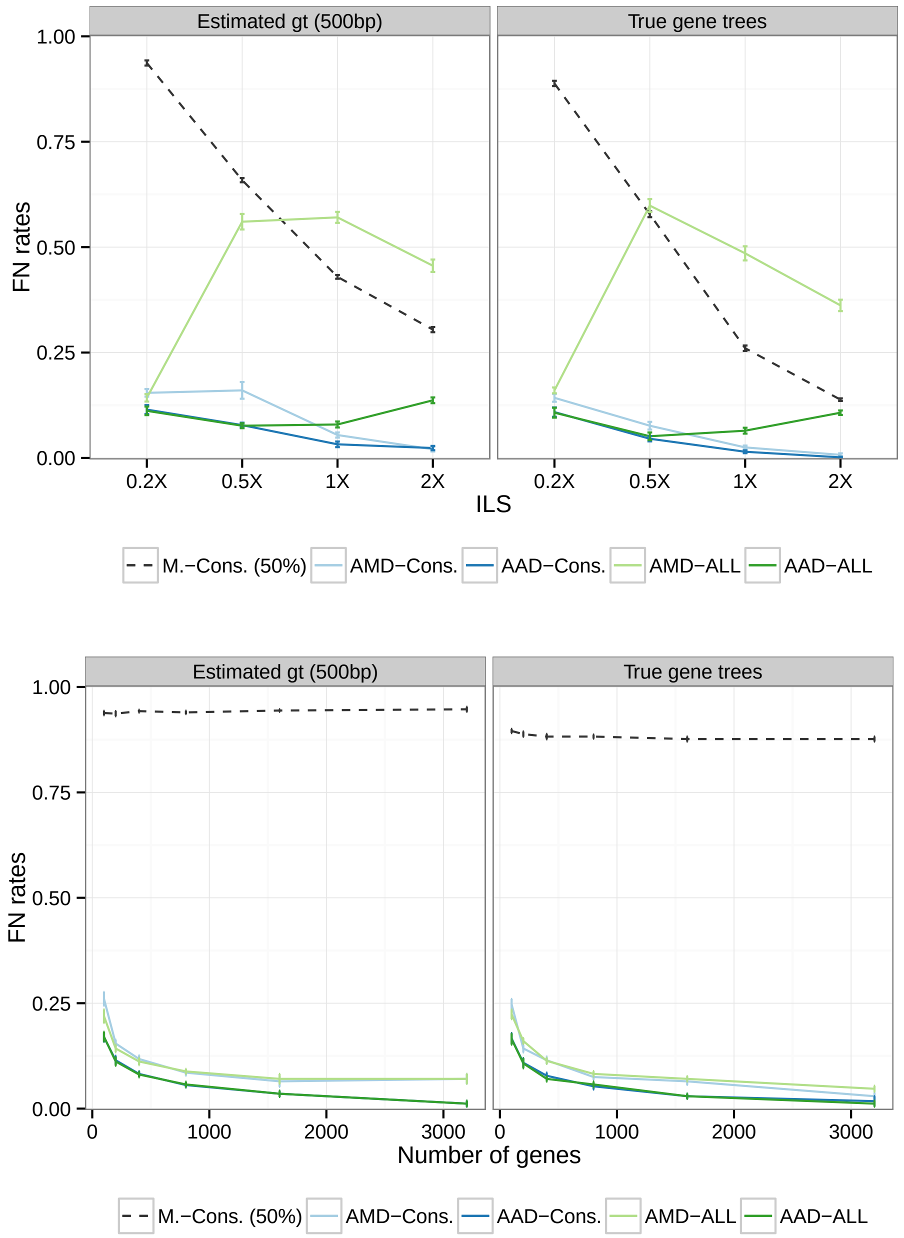

Figure S1: **Accuracy of different implementations of DISTIQUE for the Mammalian dataset.** This figure compares four versions of DISTIQUE: all-pairs-max on the consensus (AMD-Cons.), all-pairs on the consensus (AAD-Cons.), all-pairs-max on the full dataset (AMD-ALL), all-pairs on the full dataset (AAD-ALL). We also show the FN rate of the unresolved consensus tree (black dashed line). (top) number of genes: 200, varying ILS; (below) ILS: 0.2X, varying number fo genes. Mean and standard error of species tree error is shown for true and estimated gene trees (500bp alignments). With very high ILS (0.2X), the accuracy for all of the implementations of DISTIQUE are close. As ILS decreases, when DISTIQUE is applied to the entire dataset, the error goes up, which is more pronounced for all-pairs-max. We attribute this pattern to difficulties of estimating long quartet lengths. When DISTIQUE is used to resolve polytomies in the consensus tree, the accuracy improves with decreased ILS, as expected. Note that even with reduced ILS, the consensus tree on estimated gene trees misses more than 25% of branches, and leaves some polytomies for DISTIQUE to resolve.

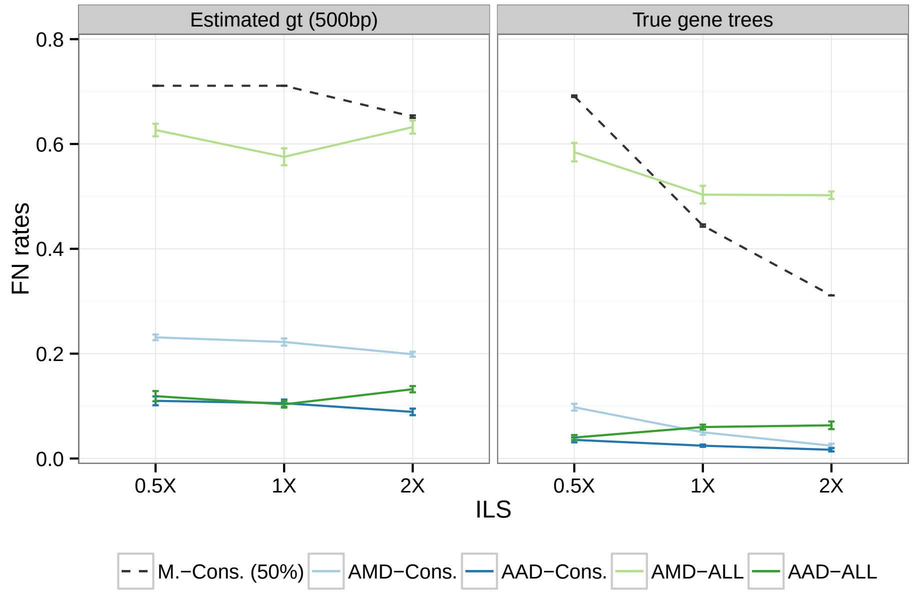

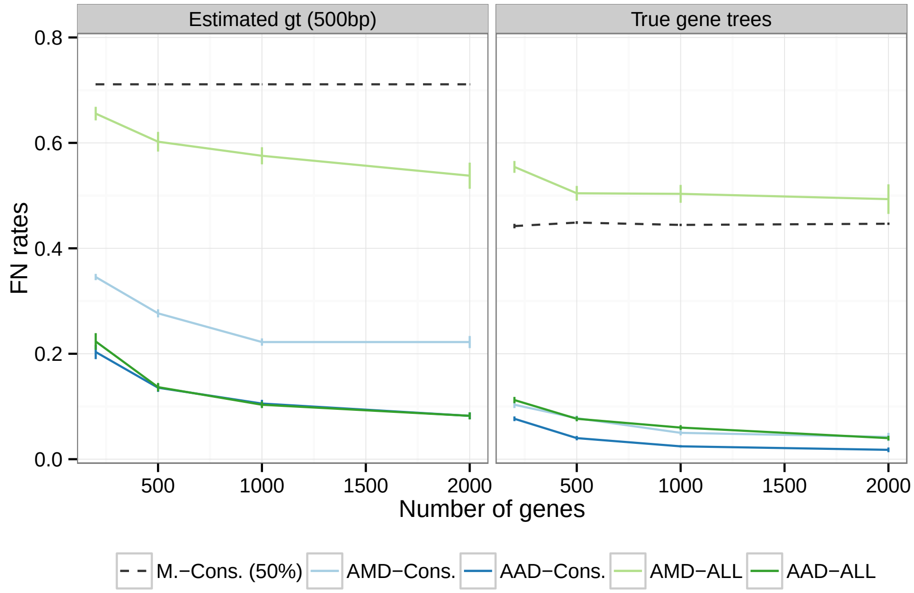

Figure S2: **Accuracy of different implementations of DISTIQUE for the Avian dataset.** This figure compares four versions of DISTIUQE on the Avian dataset. Method labels are as in Fig. S1. (top) number of genes: 1000, varying ILS; (below) ILS: 1X, varying number of genes. Mean and standard error of species tree error is shown for true and estimated gene trees (500bp alignments). Note that even with reduced ILS, the consensus tree misses more than 30% of branches, and leaves some polytomies for DISTIQUE to resolve.

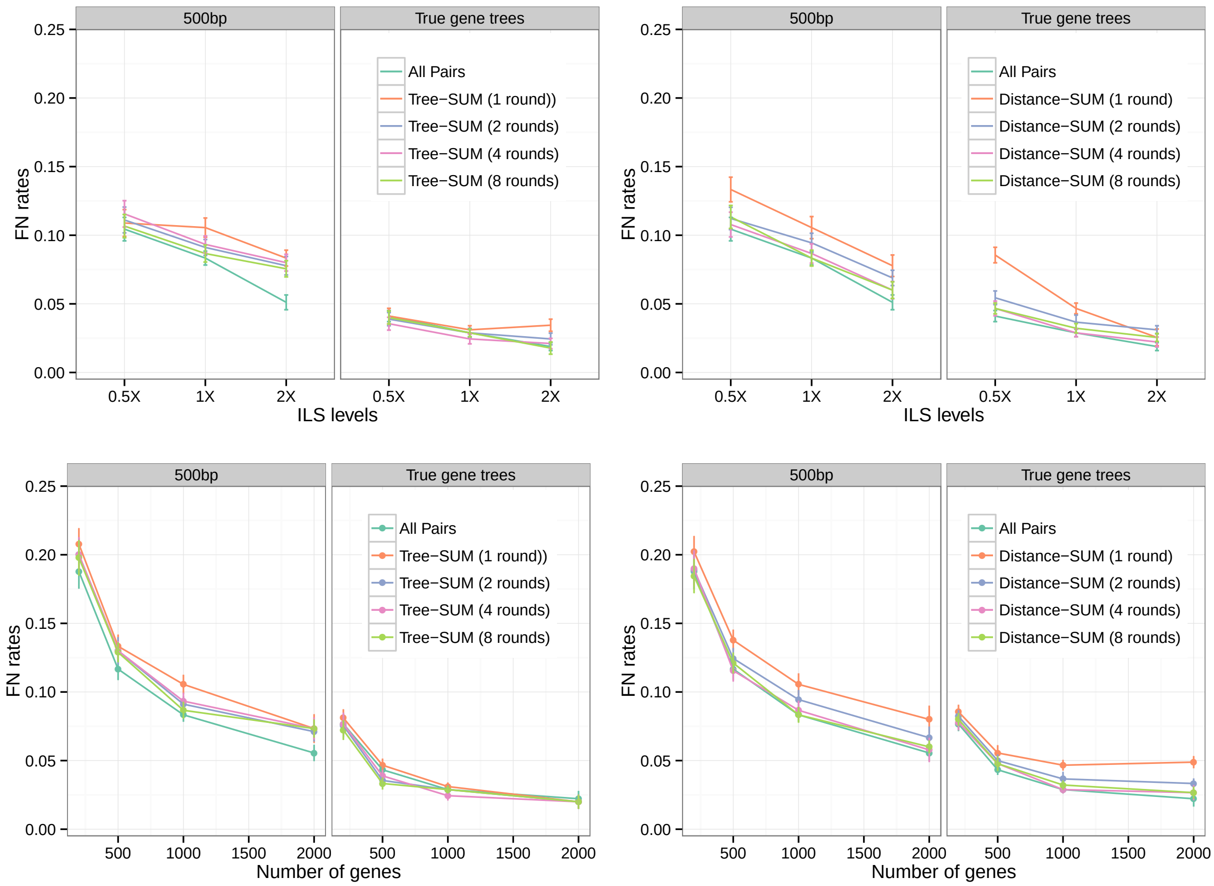

Figure S3: **Impact of number of rounds of anchor sampling on accuracy**. Dataset: Avian simulated dataset with (top) 1000 genes and varying ILS, and (bottom) 1X ILS and varying numbers of genes. The accuracy of DISTIQUE in its $\Theta(n^4)$ allPairs version is compared against $O(n^3)$ double anchored with $m = O(n)$ anchors and both tree-sum and distance-sum strategies of combining multiple anchors with various numbers of rounds of sampling. For tree-sum 2 rounds of sampling is somewhat better than one round, but beyond 2 rounds, little improvements are observed. For distance-sum, further improvements are observed as the number of rounds increases, but four rounds seems to give a reasonably good estimate without increasing the number of sampling rounds too much.

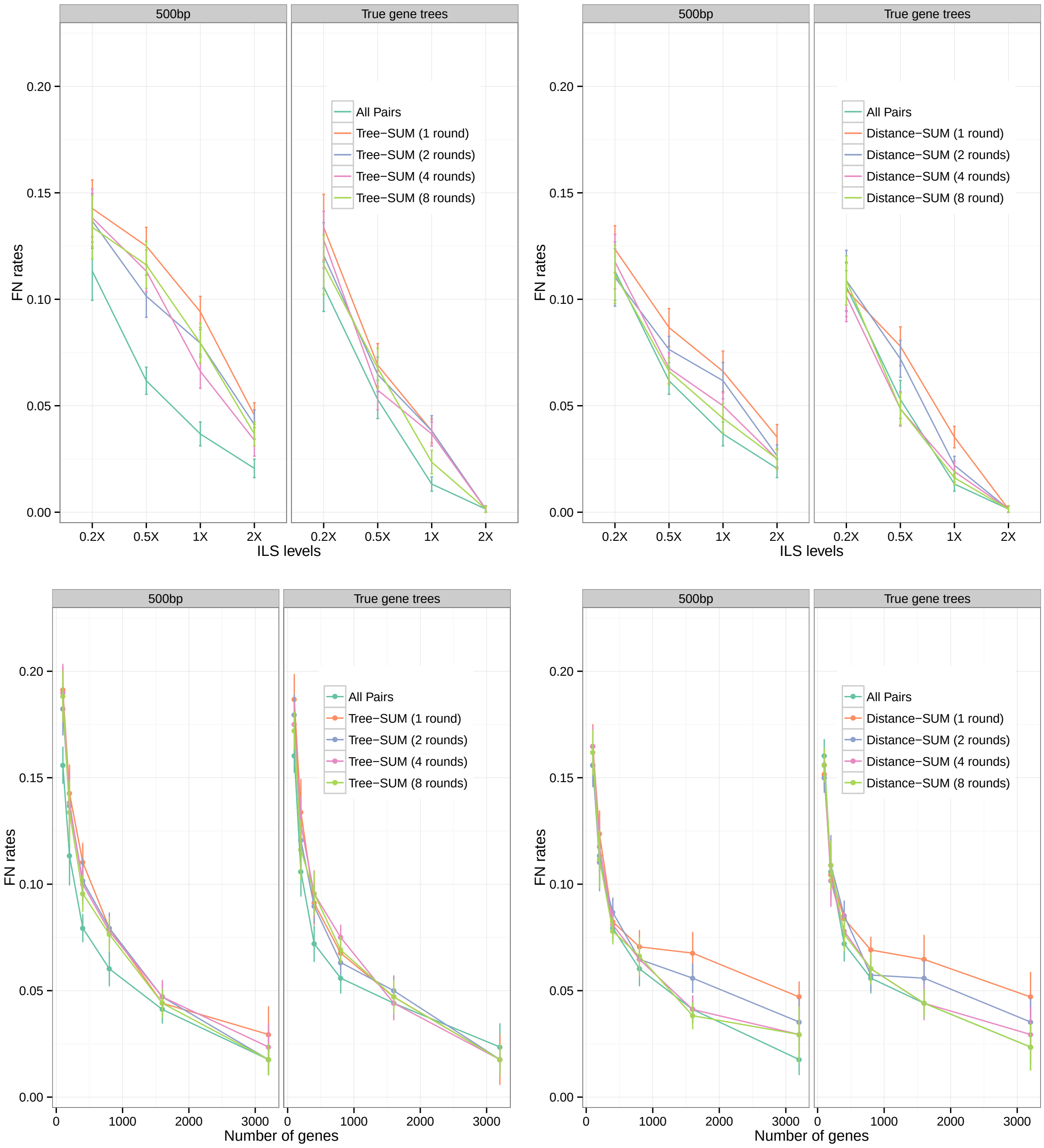

Figure S4: **Impact of number of rounds of anchor sampling on accuracy**. Dataset: Mammalian simulated dataset with (top) 200 genes and varying ILS, and (bottom) 0.2X ILS and varying numbers of genes. The accuracy of DISTIQUE in its $\Theta(n^4)$ allPairs version is compared against $O(n^3)$ double anchored with $m = O(n)$ anchors and both tree-sum and distance-sum strategies of combining multiple anchors with various numbers of rounds of sampling. For tree-sum 2 rounds of sampling is somewhat better than one round, but beyond 2 rounds, little improvements are observed. For distance-sum, further improvements are observed as the number of rounds increases, but four rounds seems to give a reasonably good estimate without increasing the number of sampling rounds too much.

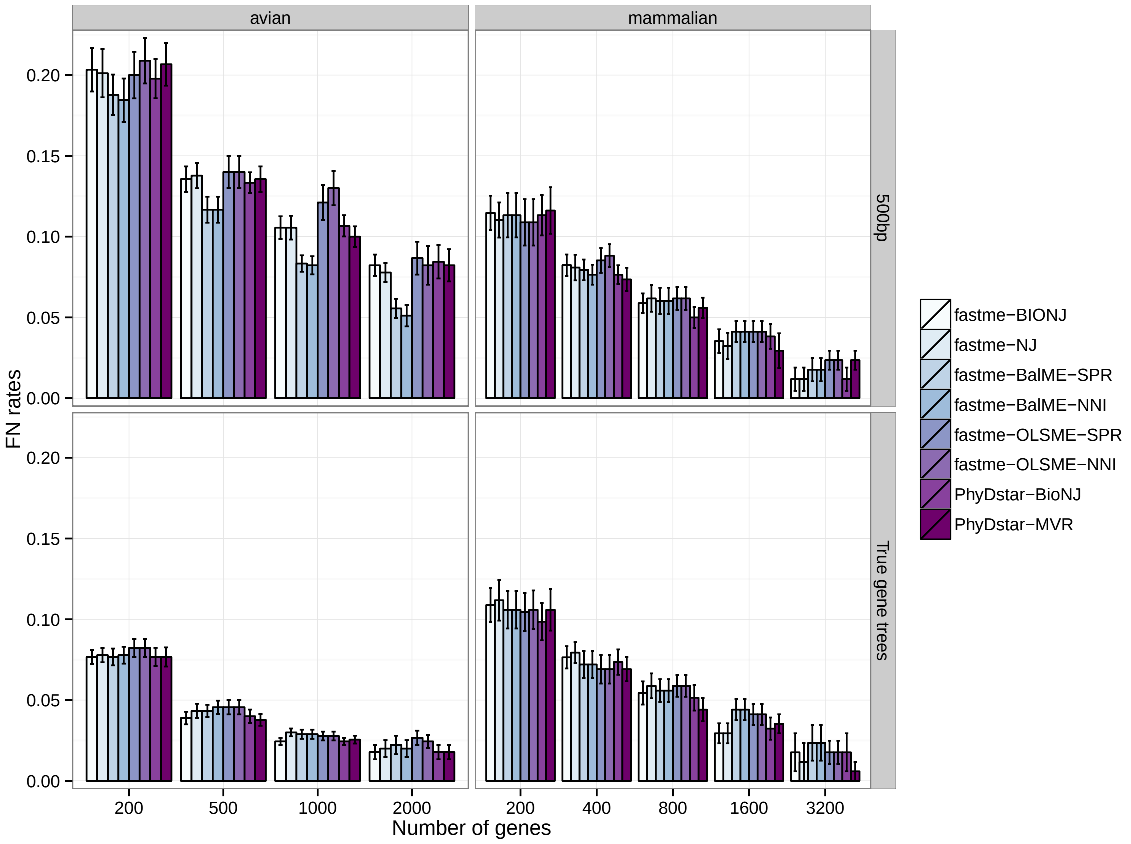

Figure S5: **Impact of distance method on accuracy**. Dataset: Mammalian and aviand simulated dataset with varying levels of ILS on both true and estimated gene trees. Each bar graph is over results from different numbers of genes, ranging between 100 and 3200 for mammalian, and 200 to 2000 for avian. The accuracy of DISTIQUE with various distance methods is used. Methods used from the FastME suit: BioNJ, Neighbor-Joining, BalME (-s), BalME (-nni), OLSME (-s), OLSME (-nni). Methods used from the PhyD* suit: BinoNJ and MVR.

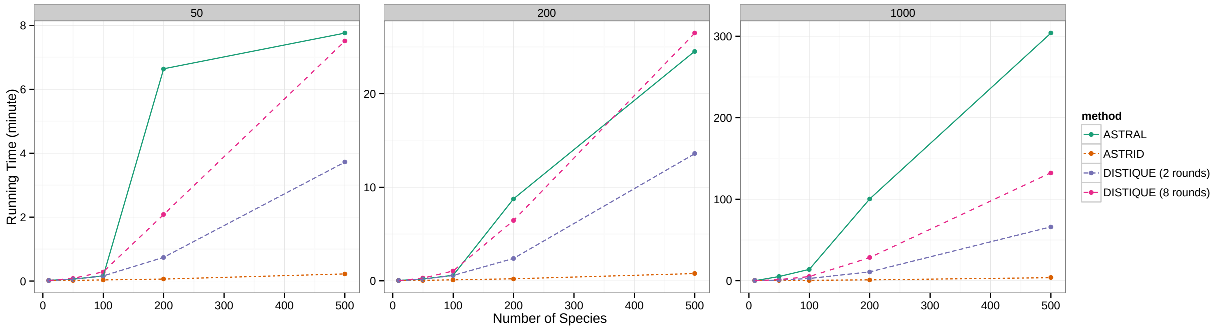

Figure S6: **Running times of DISTIQUE versus other methods for the simPhy-size dataset.** Average running times of ASTRAL, NJst, and DISTIQUE are shown in minutes for different numbers of genes (boxes).

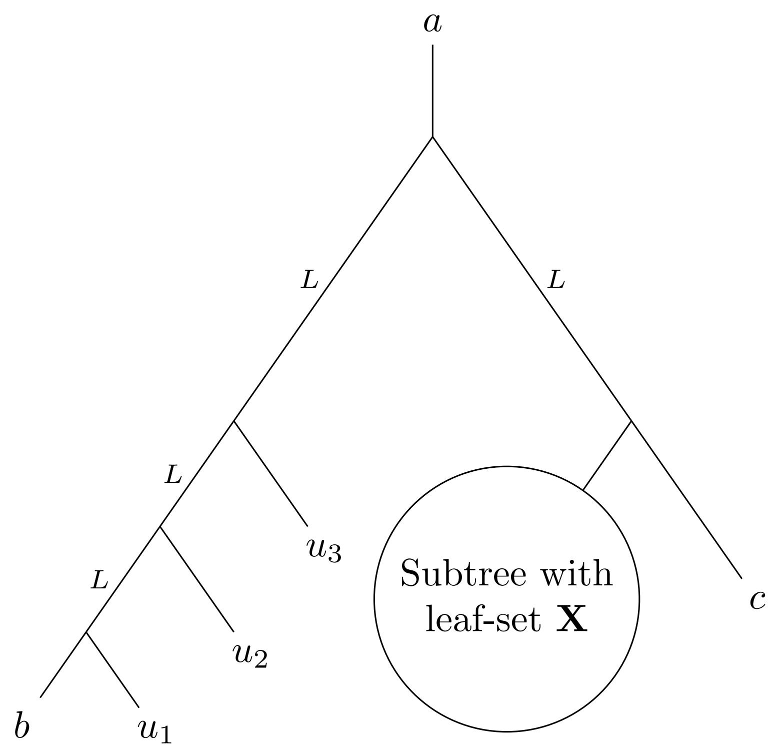

Figure S7: **An example where long branches can cause problems.** See section 2.3 for descriptions.

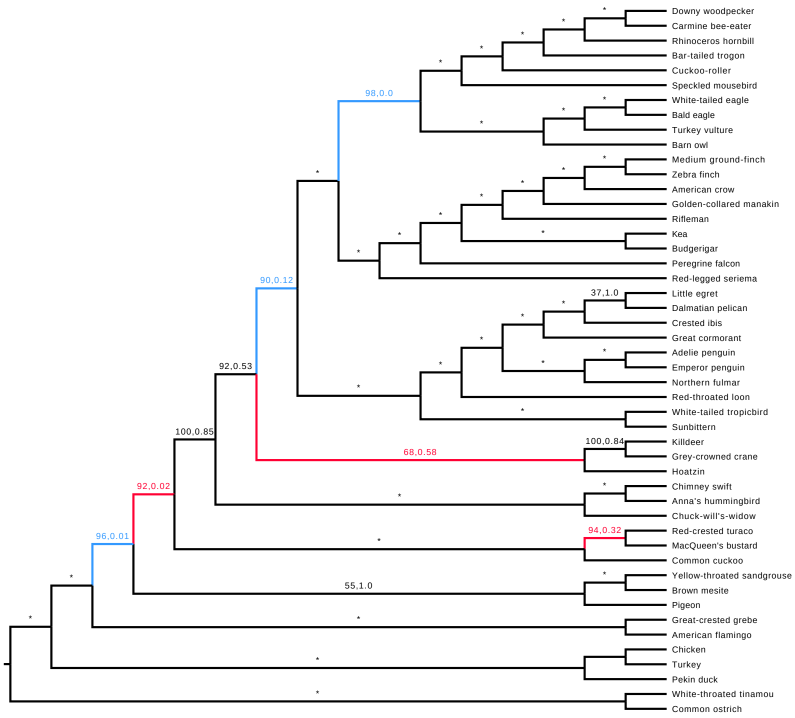

Figure S8: **Species trees generated using DISTIQUE on Avian biological dataset [1].** Branches that are different between the DISTIQUE tree and both ASTRAL and ASTRID trees are marked in red, while branches that are only different between ASTRAL and DISTIQUE are marked with blue.

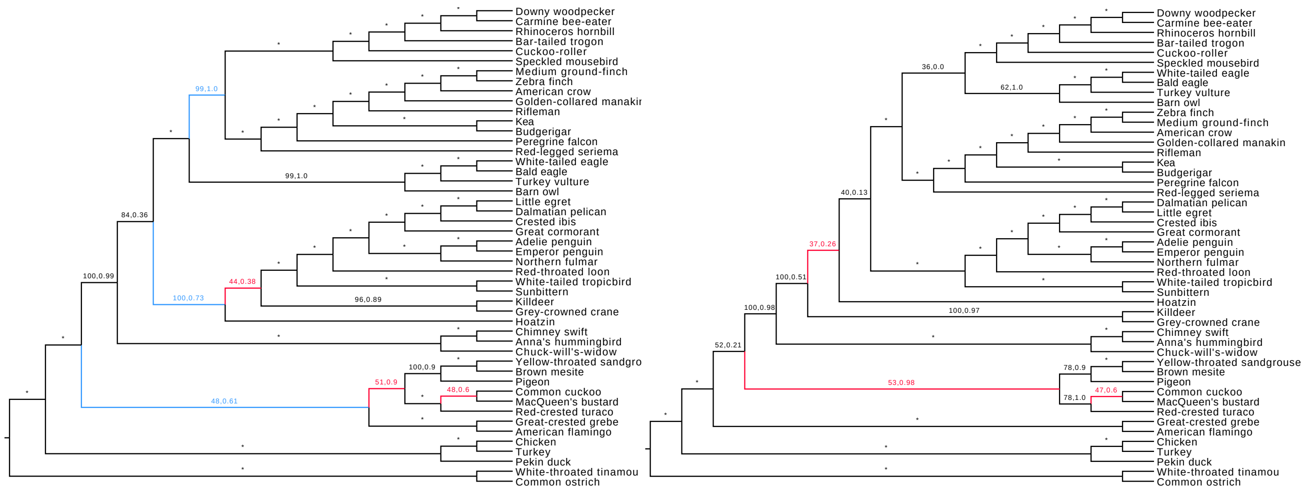

Figure S9: **Species trees generated using ASTRID (NJst) and ASTRAL on Avian biological dataset [1].** ASTRAL (left) and ASTRID (right) species trees. The branches that are different between DISTIQUE species tree and both ASTRAL and ASTRID species trees are marked with red, while branches that are only different between ASTRAL and DISTIQUE are marked with blue.

2 Supplementary theoretical results

2.1 Supplementary lemmas

Lemma S1 *Given m species trees, each on $n-2$ leaves and assuming the set of m trees is complete, and each tree is binary and correct, a compatibility supertree generates the correct tree.*

Proof. Imagine we didn't and the supertree had a wrong internal branch. For every quartet defined around that branch (selecting a leaf from each of the four sides around the branch), by completeness, there is a tree T_i that has that quartet, and because T_i is correct and binary, that quartet should be resolved correctly. Since we can argue this for all quartets around the wrong branch, we get a contradiction where the branch is wrong but all quartets around it are correct. Moreover, since our m trees are binary and complete, the supertree is binary. Imagine it wasn't and take an unresolved quartet around its polytomy; by completeness, that quartet is at least in one T_i. Thus, the supertree does not refine input tree T_i, contradicting the compatibility supertree requirement. Since the supertree has no wrong edges and is binary, it is the correct tree.

□

Lemma S2 *Given a species tree not necessarily fully resolved with n leaves, anchor-sampling strategy in Algorithm S4 yeilds at most $O(n)$ anchors per each round of anchor sampling.*

Proof. We have two types of polytomies; a polytomy is short if its degree is smaller than 5, and otherwise it is called a large polytomy. Our anchoring strategy is that per each round of sampling we have $\lceil \frac{d_i}{2} \rceil$ number of samples for a large polytomy, 6 samples for a polytomy of degree $d_i = 4$, and 10 anchors for a polytomy of degree $d_i = 5$. Note that a species has at most $n-2$ internal nodes, $n-3$ internal branches and n terminal (trivial) branches. Also, each internal branch might be adjacent to at most two polytomies.

$$\sum_{i=1}^{m} d_i \leq 2 \times (n-3) + n = 3n - 6, \tag{1}$$

where m is the number of internal nodes. In one extreme, let's consider the case where all the polytomies are large; then, the number of anchors required per each round of anchor sampling is

$$\#anchors = \sum_{i=1}^{m} \lceil \frac{d_i}{2} \rceil \leq \sum_{i=1}^{m} d_i \leq 3n - 6, \tag{2}$$

which is clearly $O(n)$. Now let's assume that all the polytomies are small. The species tree has at most $n-3$ internal branches which might be in at most two small polytomies. So we might have at most $2(n-3)$ small polytomies, then

$$\#anchors = 2(n-3) \times 10 \tag{3}$$

where 10 in above equation is the maximum number of required samples for a short polytomy. Finally, we might have a combination of small polytomies, and large polytomies and the number of required anchors is at most sum of required anchors from Equations 2, and 3, which is clearly $O(n)$. □

2.2 Difficulties with long branches and need for majority consensus

In this section, we will explain a problematic case that leads us to use a majority consensus. Figure S7 shows an unrooted species tree, with many long branches, with length L. We assume L is long enough that for our given number of gene trees, with high probability, all gene trees will be topologically identical to the species tree. Note that for any number of genes, there are branches long enough where discordance is highly unlikely (we give one example below). Thus, we assume that for branches of length L, we have zero quartet trees that conflict with them.

In our example, the distance of a to c is L and a to b is $3L$ (note that in our analyses, we only calculate branch length for internal branches). We will show that all-pairs can be misled to give a smaller distance for a to b than a to c.

Recall The definition of distance matrices:

$$D'_{uv}[a,b] = \begin{cases} \beta + \alpha.\tau(a,b,u,v) & ab.uv \notin \mathcal{Q}^T \\ \beta - f(\tau(a,b,u,v)) & ab.uv \in \mathcal{Q}^T \end{cases} \tag{4}$$

$$D'_v[a,b] = \sum_{u \in \mathcal{L}-\{a,b,v\}} D'_{uv}[a,b]. \tag{5}$$

$$D'[a,b] = \sum_{v \in \mathcal{L}-\{a,b\}} D'_v[a,b] = \sum_{u,v \in \mathcal{L}-\{a,b\}} D'_{uv}[a,b]. \tag{6}$$

$$D''[a,b] = \max_{u,v \in \mathcal{L}-\{a,b\}} \max(0, \frac{D'_{uv}[a,b]-\beta}{\alpha}). \tag{7}$$

Also recall that for coalescent-based analyses, $\beta = \ln 3$, $\alpha = 1$, $f(x) = \ln(3-2e^{-x})$, and with our use of add-half smoothing, Equation 4 simplifies to

$$D'_{uv}[a,b] = -\ln \overline{p}(ab.uv) = -\ln(\frac{freq(ab.uv)+0.5}{n+1.5}) \tag{8}$$

where n is the number of genes, and $freq(ab.uv)$ is the number of genes with induced quartet topology $ab.uv$. Using Equations 6 and 8 and considering all selections of anchors u and v, we have

$$\begin{aligned} D'[a,b] =& -\binom{3}{1}.\binom{|X|+1}{1}.\ln(\frac{0.5}{n+1.5}) - \binom{|X|+1}{2}\ln(\frac{n+0.5}{n+1.5}) \quad -\binom{3}{2}\ln(\frac{0.5}{n+1.5}) \\ \approx& -3(|X|+2)\ln(\frac{0.5}{n+1.5}) \end{aligned} \tag{9}$$

The first term comes from choosing one anchor from $\{u_1, u_2, u_3\}$, and choosing the other anchors from $\{c\} \cup X$. In these cases, a and b are further from each other than the anchors, and because of our assumption about L, the frequency of $ab.uv$ is expected to be zero. The second term comes from choosing both anchors from $\{c\} \cup X$; for these, the frequency of $ab.uv$ is expected to be n. Finally, the last term comes from choosing both anchors from the set $\{u_1, u_2, u_3\}$, where once again, the expected frequency of $ab.uv$ is zero for long enough L, leading to the use of the pseudo count. For large enough n, we can approximate $\ln(\frac{n+0.5}{n+1.5}) \approx 0$.

The same thing could be written for a and c:

$$\begin{aligned} D'[a,c] =& -\binom{4}{1}.\binom{|X|}{1}\ln(\frac{0.5}{n+1.5}) - \binom{|X|}{2}\ln(\frac{n+0.5}{n+1.5}) \quad -\binom{4}{2}\ln(\frac{n+0.5}{n+1.5}) \\ \approx& -4(|X|)\ln(\frac{0.5}{n+1.5}) \end{aligned} \tag{10}$$

The first term comes from choosing an anchor from of the $\{u_1, u_2, u_3, b\}$, and the other anchor from X; here, for long enough L, frequency of $ab.uv$ would be expected to be zero. The second term comes from choosing both anchors from X, and the last term comes from choosing both anchors from $\{u_1, u_2, u_3, b\}$; in these cases we expect the frequency of $ab.uv$ to be n.

Comparing the Equations 9 and 10, for $|X| > 2$, the distance between a and b would be smaller than the distance between a and c. This clearly is in contradiction to our tree, so DISTIQUE without the use of majority consensus, in this case, becomes misleading. However, note that all branches with length L are assumed to generate no discordance, and thus will be in the final tree.

Large L: Assume that $L = 16$ (in coalescent units) and we have 1000 gene trees. The probability of having only topologies that agree with the species tree in all 1000 gene trees is $(1-2/3e^{-16})^{1000} = 0.99993$. Thus, we expect all n gene trees to have that topology with very high probability. The probability of having only the species tree topology for branches of length $2L$ and $3L$ is even larger.

2.3 Computing pseudo-counts for allpairs-max

In allpairs-max methods, in case of zero frequencies for some quartet topologies, without changing the definition of the pseudo-count, the relative information about quartets would be lost. For example, assume we have topology $ab.cz$ with long internal branch length, like 16 as mentioned, and $ab.dz$ with internal branch length of 20 (longer than the previous length), and no sample for none of the topologies that contradict the species tree. In this case, the distance between a, and c is equal to the distance of a, and d which is $\ln \frac{0.5}{n+1.5}$, where n is the number of samples. So the relative information is lost. In order to avoid this problem, the definition of pseudo count in allpairs-max is slightly changed. In order to have a pseudo count that could capture the relative distances, first, the number of zero quartet topologies are counted. This is called n^0_{ab}. The pseudo count, in this case, is defined as:

$$\ln \prod_{i=1}^{n^0_{ab}} \frac{0.5}{k_i + 1.5} \tag{11}$$

Where 0.5 comes from our add half estimator, is probability of zero frequencies, and k_i is the number of samples for quartet topology of i.

2.4 Tree inference algorithms

Let $\mathcal{D}$ denote the input data (e.g., for a summary method, the set of input gene trees, $\mathcal{G}$, was the input data). Regardless of the nature of $\mathcal{D}$, we require having a quartet estimator:

Quartet estimator A quartet estimator $\theta^{\mathcal{D}}(Q)$ is a function that given a quartet of leaves $Q = \{a, b, c, d\}$, uses $\mathcal{D}$ to estimate the quartet tree topology and the quartet length $\tau(Q)$. A quartet estimator is statistically consistent if, as the size of $\mathcal{D}$ increases, the estimated quartet topology and length both converge in probability to correct values.

Statistically consistent quartet estimators can be designed for various types of data. For example, the four point condition can estimate quartet trees from sequence data [6], and the log-det method gives a model-based way of estimating corrected branch lengths [7]. As we will show, DISTIQUE uses a consistent coalescent-based quartet estimator.

Given a consistent quartet estimator $\theta^{\mathcal{D}}(.)$, Theorems 1 and 2 define a family of statistically consistent phylogenetic reconstruction methods that range in running time between $\Theta(n^2)$ and $\Theta(n^4)$. Algorithm S1 shows general forms of these algorithms. All-pairs and all-pairs-max, which are the simplest methods in this family, use equations (6) and (7) to compute the distance matrix. They then compute the tree using neighbor joining [8–10] (but any consistent distance method with a safety radius [10] could be used).

Algorithm S1 Anchored quartet-based algorithms. $\theta^{\mathcal{D}}(Q)$ is a quartet estimator and returns the quartet topology and length $\tau(Q)$. α and β are constants, and $f(x)$ is a monotonically increasing function bounded above by β for positive x (i.e., $0 < f(x) < \beta$ for $x > 0$). *Anchors*(.) uses some strategy to select a subset of all possible anchor pairs.

function ANCHORS($\mathcal{L}$)
  return a set of anchor pairs

function $D'_{uv}(a,b)$
  $(t,d) \leftarrow \theta^{\mathcal{D}}(a,b,u,v)$
  if $t = ab.uv$ **then return** $\beta - f(d)$
  else return $\beta + \alpha.d$

function $D'(u,v)$
  $D'_{uv} \leftarrow 0_{n-2\times n-2}$
  for $\{a,b\} \subset \mathcal{L} - \{u,v\}$ **do**
    $D'_{uv}[a,b] \leftarrow D'_{uv}(a,b)$
  return $D'_{u,v}$

function ALL-PAIRS-MAX($\mathcal{D}$)
  $D'' \leftarrow 0_{n\times n}$
  for $\{a,b\} \subset \mathcal{L}$ **do**
    for $\{u,v\} \subset \mathcal{L} - \{a,b\}$ **do**
      if $D''[a,b] < D'_{uv}(a,b)$ **then**
        $D''[a,b] \leftarrow D'_{uv}(a,b)$
  return NeighborJ(D'')

function ALL-PAIRS($\mathcal{D}$)
  $D' \leftarrow 0_{n\times n}$
  for $\{u,v\} \subset \mathcal{L}$ **do**
    $D' \leftarrow D' + D'(u,v)$
  return NeighborJ(D')

function DISTANCE-SUM($\mathcal{D}$)
  $\mathcal{M} \leftarrow []$
  for $\{u,v\} \subset$ Anchors($\mathcal{L}$) **do**
    $\mathcal{M} \leftarrow [\mathcal{M}, D'(u,v)]$
  $DS \leftarrow$ average($\mathcal{M}$)
  return NeighborJ(DS)

function TREE-SUM($\mathcal{D}$)
  $\mathcal{T} = []$
  for $\{u,v\} \subset$ Anchors($\mathcal{L}$) **do**
    $\mathcal{T} \leftarrow [\mathcal{T}$,NeighborJ($D'(u,v)$)]
  return SuperTree($\mathcal{T}$)

Theorem S1 *All-pairs and all-pairs-max phylogenetic reconstruction methods shown in Algorithm S1 are statistically consistent.*

Proof (sketch). Since $\theta^{\mathcal{D}}(.)$ is assumed statically consistent, in limit, it will return the correct quartet topology with arbitrarily high probability, and its estimates of quartet lengths can be made arbitrarily close to true values with any desired probability. From this and Theorems 1 and 2, it follows that distance matrices used in AllPairs and AllPairsMax are arbitrarily close to additive, with high probability. The proof follows from the statistical consistency of neighbor joining for distance matrices that are within a safety radius of additivity [10]. □

Algorithms Distance-sum and Tree-sum use a subset of all $\binom{n}{2}$ anchors, combining the results from multiple anchors using methods we described in Section 3.2.3 of the main paper. Both distance-sum and tree-sum methods first select m pairs of anchors with a criterion of choice (e.g., those mentioned for DISTIQUE in Section 3.2.2 of the main paper and detailed in Algorithm S4). For each anchor pair $\{u,v\}$, a double anchored distance matrix on $\mathcal{L} - \{u,v\}$ is computed. Tree-sum computes m trees, each on $n-2$ leaves using neighbor joining [8], and then combines the m trees using a supertree method (e.g., SuperFine [11] or MRL [12]). Distance-sum combines distance matrices by averaging them, ignoring missing values. We have not been able to prove consistency for the distance-sum strategy, but tree-sum can be proved consistent with an appropriate choice of the supertree method. Recall a *compatibility* supertree refines all its inputs in the output when the input trees are compatible. We define a set of trees *complete* if every quartet of leaves appears in at least one tree.

Theorem S2 *The tree-sum phylogenetic reconstruction method shown in Algorithm S1 is statistically consistent if used with a compatibility supertree and when the anchor selection generates a complete set of m trees.*

Proof. By Theorem 1 and arguments similar to those used for Theorem S1, each tree $T_1 \dots T_m$

on $n-2$ leaves is a statistically consistent estimate; thus, for any $\epsilon' < 1$, there is a dataset size such that each T_i is correct with probability $1-\epsilon'$. Taking the largest of these dataset sizes, with probability at least $(1-\epsilon')^m$, every T_i is correct. By setting $\epsilon' < 1-(1-\epsilon)^{\frac{1}{m}}$ we argue that for any ϵ, there is a dataset size where all T_is are correct with probability at least $1-\epsilon$. By Lemma S1 the use of a compatibility supertree method and the completeness of m binary and correct trees guarantee that the supertree is the correct tree. □

Combining these algorithms with our approaches for dealing with long branches (i.e., the use of a consensus tree) results in a somewhat more complicated algorithm. Algorithm S2 shows the detailed steps for our default distance-sum approach.

A note on branch lengths: Anchored distances (when computed exactly) set the length of terminal branches to zero. A constant can be added to all distances for inference methods that expect non-zero terminal lengths. AllPairsMax returns statistically consistent estimates of *internal* branch lengths, but branch lengths from our other methods should be ignored.

Table S4: **Description of variables and what they contain in Algorithm S2**

Variable Name	Variable Type	What Contains
$\mathcal{G}$	list of trees	list of gene trees
$sRnd$	Integer number	# rounds of sampling
$consTree$	tree	consensus tree
$smallPoly$	list	list of small polytomies (degree ≤ 5)
$largePoly$	list	list of large polytomies (degree > 5)
$anchPairsSmallPoly$	dictionary of lists	sampled pairs of anchors around small polytomies
$allAnchPairs$	list	list of all sampled pairs of anchors
$clustPoly$	dictionary of matrices	matrix of taxa under clusters around polytomies.
c_1	index	index of the cluster that the selected anchor belongs to
c_2	index	index of the cluster that the selected anchor belongs to
C	matrix	$C[i][j]$ stores number of quartets of the form $c_1c_2\|ij$
D	matrix	$D[i][j]$ stores maximum possible quartets of the form $c_1c_2\|ij$
$anchDist$	matrix	$anchDist[i][j]$ stores distance between cluster i, and j
$anchNorm$	matrix	$anchNorm[i][j]$ is 1, if $i \notin \{c_1, c_2\}$, and $j \notin \{c_1, c_2\}$. Otherwise is 0.
$totalAnchDist$	matrix	$totalAnchDist[i]$ contains sum of distance matrices for polytomy i
$totalAnchNorm$	matrix	stores # of times distance between j and z for polytomy i estimated
$totalAnchDist$	matrix	averaged distance between i and j for a polytomy
$spTree$	list of trees	$spTree[node]$ estimated species tree for polytomy around $node$
$speciesTree$	tree	Estimated species tree

Algorithm S2 DISTIQUE distance sum algorithm. $\mathcal{G}$ is the set of input gene trees. *sRnd* determines the number of rounds of sampling anchors. The description of variables are provided in Table S4. Also the details of functions anchoredFrequencies, distanceAroundPolyNodes, and getClusterAnch are provided in Algorithm S3.

function DISTIQUE-DISTANCE-SUM($\mathcal{G}$, *sRnd*)
  $consTree \leftarrow$ consensus($\mathcal{G}$)
  $consTree \leftarrow$ labelNodes($consTree$)
  $(smallPoly, largePoly, clustPoly) \leftarrow$ findPolytomies($conTree$)
  $polyNodes \leftarrow smallPoly \cup largePoly$
  $(anchPairsSmallPoly, allAnchPairs) \leftarrow$ sampleAnchors($polyNodes, clustPoly, sRnd$)
  for all $node \in largePoly$ **do**
    for all $anchPair \in allAnchPairs$ **do**
      $(c_1, c_2) \leftarrow$ getClusterAnch($anchPair, clustPoly[node]$)
      if $c_1 \neq c_2$ **then**
        $(C, D) \leftarrow$anchoredFrequencies($anchPair, \mathcal{G}, clustPoly[node]$)
        $(anchDist, anchNorm) \leftarrow$distanceAroundPolyNode($anchPair, C, D, clustPoly[node]$)
        $totalAnchDist[node] \leftarrow totalAnchDist[node] + anchDist$
        $totalAnchNorm[node] \leftarrow totalAnchNorm[node] + anchNorm$
  for all $node \in smallPoly$ **do**
    for all $anchPair \in anchPairsSmallPoly[node]$ **do**
      $(C, D) \leftarrow$anchoredFrequencies($anchPair, \mathcal{G}, clustPoly[node]$)
      $anchDist \leftarrow$distanceAroundPolyNode($anchPair, C, D, clustPoly[node]$)
      $totalAnchDist[node] \leftarrow totalAnchDist[node] + anchDist$
      $totalAnchNorm[node] \leftarrow totalAnchNorm[node] + anchNorm$
  for all $node \in totalAnchDist$ **do**
    $totalAnchDist[node] \leftarrow totalAnchDist[node]/totalAnchNorm[node]$
    $spTree[node] \leftarrow$ NeighborJ($totalAnchDist[node]$)
  $speciesTree \leftarrow consTree$
  for all $node \in postorderTraverse(speciesTree)$ **do**
    if $node \in spTree$ **then**
      $speciesTree \leftarrow$resolvePoly($speciesTree, spTree[node]$)
  return $speciesTree$

Algorithm S3 This algorithm describes details of functions anchoredFrequency, distanceAroundPolyNodes, and getClusterAnch used in Algorithm S2. anchoredFrequency returns frequency of observing quartets of the form $c_1c_2|uv$, where c_1, and c_2 are clusters corresponding to chosen anchors around one polytomy. distanceAroundPolyNode returns distance between cluster u, and v. These distances will be used to infer a resolution of polytomy using phylogeny estimation methods. getClusterAnch determines which clusters anchors belong to.

```
function anchoredFrequencies(anchPair, 𝒢, clusters)
  (c_1, c_2) ← getClusterAnch(anchPair, clusters)
  C ← []
  D ← []
  for {i, j} ∈ twoSubsets({1, 2, ..., |clusters|) do
    if i ∈ {c_1, c_2} ∨ j ∈ {c_1, c_2} then
      D[i][j] ← 0
    else
      D[i][j] ← |clusters[i]| × |clusters[j]| × |𝒢| + 1.5      ▷ 1.5 is for smoothing by add-half
      C[i][j] ← 0.5                                           ▷ 0.5 is for smoothing using add-half estimator
  for g ∈ 𝒢 do
    g ← reroot(g, anchPair[0])
    node ← anchPari[1]
    repeat
      pre ← node
      node ← node.parent
      children ← node.children
      children ← children − pre
      for k ∈ {1, 2, ..., |children|} do
        C_k ← getTaxaUnderClusters(children[k], clusters)
        for {i, j} ∈ twoSubsets({1, 2, ..., |clusters|) do
          C[i][j] ← C[i][j] + C_k[i] × C_k[j]
        if |neighbors| ≥ 2 then
          for z ∈ {k + 1, ..., |children|} do
            C_z ← getTaxaUnderClusters(children[z], clusters)
            for {i, j} ∈ twoSubsets({1, 2, ..., |clusters|) do
              D[i][j] ← D[i][j] − C_z[i] × C_k[j] − C_z[j] × C_k[i]
    until node.parent == anchPair[1]
  return (C, D)
function distanceAroundPolyNode(anchPair, C, D, clusters)
  for {i, j} ∈ twoSubsets({1, 2, ..., |clusters|) do
    if ¬(anchPair[0] ∈ clusters[i] or anchPair[1] ∈ clusters[i]) then
      if ¬(anchPair[0] ∈ clusters[j] ∨ anchPair[1] ∈ clusters[j]) then
        anchDist[i][j] ← − ln (C[i][j]/D[i][j])
        anchNorm[i][j] ← 1
        anchDist[j][i] ← − ln (C[i][j]/D[i][j])
        anchNorm[j][i] ← 1
  return (anchDist, anchNorm)
function getClusterAnch(anchPair, clusters)
  c ← {}
  for i ∈ {1, 2, ..., length(clusters)} do
    if anchPair[0] ∈ clusters[i] then
      c ← c ∪ clusters[i]
  return c
```

Algorithm S4 Sample anchors algorithm. The variable *polyNodes* contains the polytomies in the tree, and *clustPoly* contains labeled nodes around each polytomy and the list of taxa under each cluster. Also the variable *sRnd* determines the number of sampling rounds around each polytomy.

```
function sampleAnchors(polyNodes, clustPoly, sRnd)
  allAnchors ← []
  for all node ∈ polyNodes do
    clusters ← clustPoly[node]
    if length(clustPoly[node]) ≥ 6 then
      largeAnch ← sampleAnchLargePoly(clusters, sRnd)
      allAnchors ← allAnchors ∪ largeAnch
    else
      anchSmallPoly[node] ←sampleAnchSmallPoly(clusters)
      allAnchors ← allAnchors ∪ anchSmallPoly[node]
  return (anchSmallPoly, allAnchors)
function sampleAnchLargePoly(cluster, sRnd)
  i ← 0
  anchPairs ← []
  C ← {1, 2, . . . ,length(cluster)}
  while i < smpRnd do
    setC ← C
    while length(setC)> 0 do
      if length(setC)> 1 then
        {u, v} ← randChooseTwoLeaves(setC)
      else
        {u} ← randChooseLeave(C − setC)
        {v} ← setC
      {taxon1} ← randChooseLeave(cluster[u])
      {taxon2} ← randChooseLeave(cluster[v])
      anchPairs ← anchPairs ∪ {(taxon1, taxon2)}
      setC ← setC − {u, v}
  return anchPairs
function sampleAnchSmallPoly(cluster)
  anchPairs ← []
  for i ∈ {0, 1, . . .,length(cluster)} do
    for j ∈ {i + 1, . . .,length(cluster)} do
      {taxon1} ←randChooseLeave(cluster[i])
      {taxon2} ←randChooseLeave(cluster[j])
      anchPairs ← anchPairs ∪ {(taxon1, taxon2)}
  return anchPairs
```

3 Commands and version numbers

We used ASTRAL version 4.7.8 to find the species trees from gene trees:

```
java -Xmx2000M -jar astral.4.7.8.jar -i [GENE TREES] -o [OUTPUT SPECIES TREE]
```

The ASTRID (NJst) results were produced using the following command:

```
python ASTRID.py -i [GENE TREES] -m [fastme2] -o [OUTPUT SPECIES TREE] -c
[CACHE]
```

CACHE is the distance matrix produced by ASTRID.

For DISTIQUE-allpairs we used the following command:

```
python distique.py -a [mean] -g [GENE TREES] -m [prod] -o [OUTPUT DIR] -t[1]
```

For DISTIQUE-allpairs-max we used the following command:

```
python distique.py -a [mean] -g [GENE TREES] -m [min] -o [OUTPUT DIR] -t[2]
```

Here the flag a specifies which averaging method to use the partial quartet tables from complete quartet tables around each polytomy.

For tree-sum we used the following command:

```
python distique.py -g [GENE TREES] -o [OUTPUT DIR] -n [# rounds of anchoring]
-t [4]
```

For distance-sum we used the following command:

```
python distique.py -g [GENE TREES] -o [OUTPUT DIR] -n [# rounds of anchoring]
-t [3]
```

Finally the flag t determines which method to use for inferring.

For comparison and computing false negative missing branches we used the command available at `https://github.com/smirarab/global/tree/master/src/shell`:

```
compareTrees.missingBranch [SPECIES TREE] [ESTIMATED SPECIES TREE ]
```

References

[1] Tae Kun Seo. Calculating bootstrap probabilities of phylogeny using multilocus sequence data. *Molecular Biology and Evolution*, 25(5):960–971, 2008.

[2] Jed Chou, Ashu Gupta, Shashank Yaduvanshi, Ruth Davidson, Mike Nute, Siavash Mirarab, and Tandy Warnow. A comparative study of SVDquartets and other coalescent-based species tree estimation methods. *BMC Genomics*, 16(Suppl 10):S2, 2015.

[3] Siavash Mirarab, Md. Shamsuzzoha Bayzid, and Tandy Warnow. Evaluating Summary Methods for Multilocus Species Tree Estimation in the Presence of Incomplete Lineage Sorting. *Systematic Biology*, 65(3):366–380, 2016.

[4] Siavash Mirarab, Md. Shamsuzzoha Bayzid, Bastien Boussau, and Tandy Warnow. Statistical binning enables an accurate coalescent-based estimation of the avian tree. *Science*, 346(6215), 2014.

[5] Md Shamsuzzoha Bayzid, Siavash Mirarab, Bastien Boussau, and Tandy Warnow. Weighted Statistical Binning: Enabling Statistically Consistent Genome-Scale Phylogenetic Analyses. *PLoS ONE*, 10(6):e0129183, 2015.

[6] Y Benjamini and Y Hochberg. Controlling the false discovery rate: a practical and powerful approach to multiple testing. *Journal of the Royal Statistical Society*, 57:289–300, 1995.

[7] Michael Steel. Recovering a tree from the leaf colourations it generates under a Markov model. *Applied Mathematics Letters*, 7(2):19–23, 1994.

[8] N Saitou and Masatoshi Nei. The neighbour-joining method: a new method for reconstructing phylogenetic trees. *Molecular Biology and Evolution*, 4(4):406–425, 1987.

[9] J A Studier and K J Keppler. A note on the neighbor-joining algorithm of Saitou and Nei. *Molecular Biology and Evolution*, 5(6):729–31, 1988.

[10] K. Atteson. The Performance of Neighbor-Joining Methods of Phylogenetic Reconstruction. *Algorithmica*, 25(2-3):251–278, 1999.

[11] M Shel Swenson, Rahul Suri, C Randal Linder, and Tandy Warnow. SuperFine: fast and accurate supertree estimation. *Systematic Biology*, 61(2):214–27, 2012.

[12] Nam Nguyen, Siavash Mirarab, and Tandy Warnow. MRL and SuperFine+MRL: new supertree methods. *Algorithms for Molecular Biology*, 7(1), 2012.
